# Supplementary material for: A Series of Green Oxovanadium(IV) Precatalysts with O, N and S Donor Ligands in a Sustainable Olefins Oligomerization Process
Source: Molecules. 2022 Nov 19;27(22):8038. doi: 10.3390/molecules27228038 (PMC9694032; doi:10.3390/molecules27228038)
Supplement: Supplementary file 1 [file molecules-27-08038-s001.zip › molecules-2035465-supplementary.pdf]

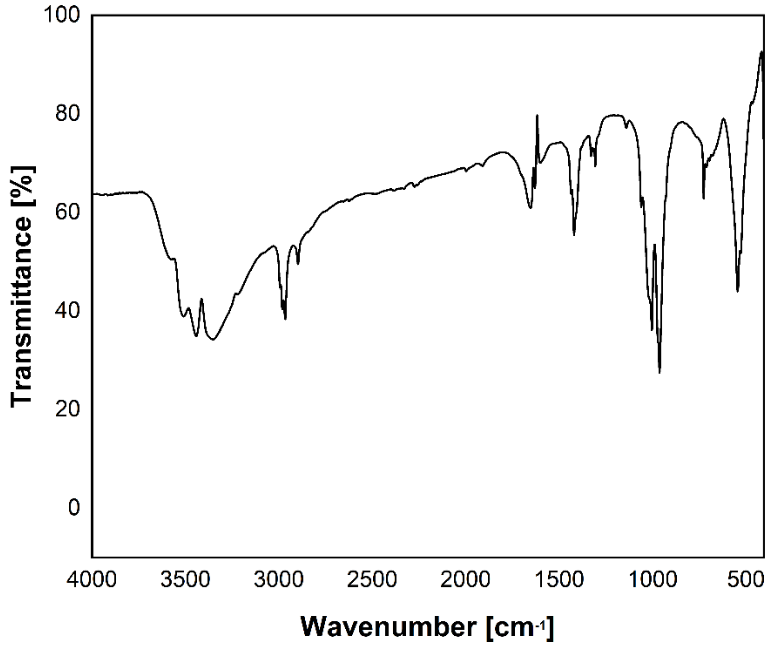

Figure S1. The results of FTIR spectroscopy for the products of oligomerization 3-buten-2-ol obtained using [VO(TDA)(phen)] • 1.5 H_2O.

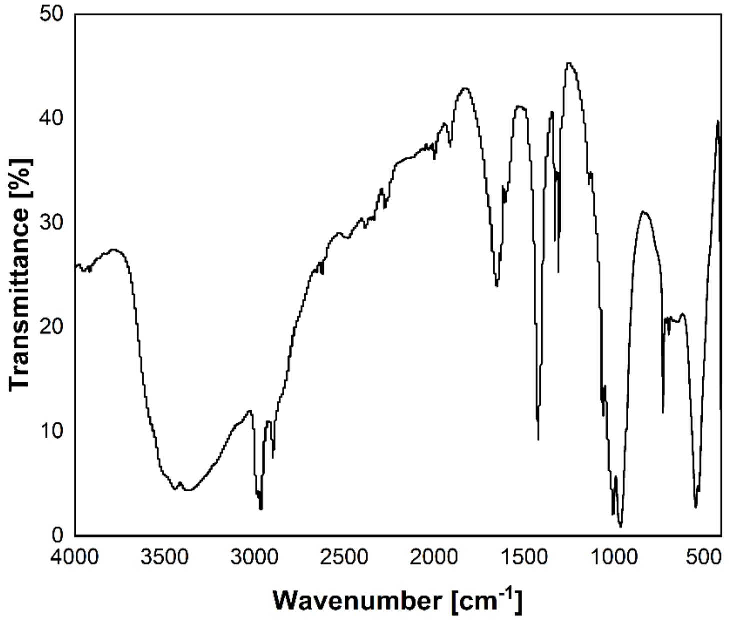

Figure S2. The results of FTIR spectroscopy for the products of oligomerization 3-buten-2-ol obtained using [VOO(dipic)(2-phepyH)] • H_2O.

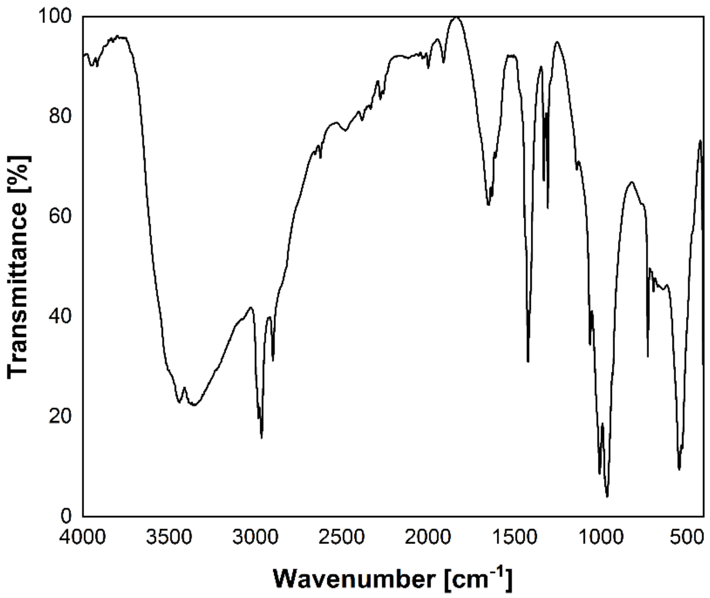

Figure S3. The results of FTIR spectroscopy for the products of oligomerization 3-buten-2-ol obtained using [VO(dipic)(dmbipy)] • 2 H_2O.

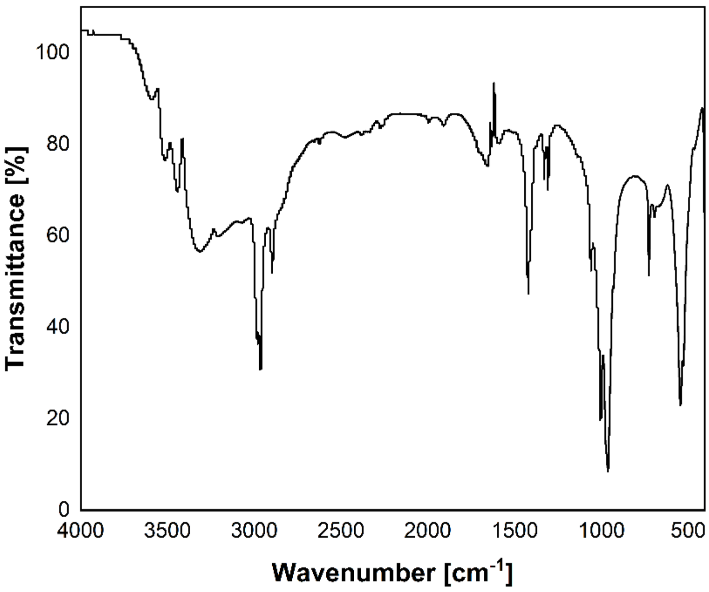

Figure S4. The results of FTIR spectroscopy for the products of oligomerization 3-buten-2-ol obtained using [VO(ODA)bipy] • 2 H_2O.

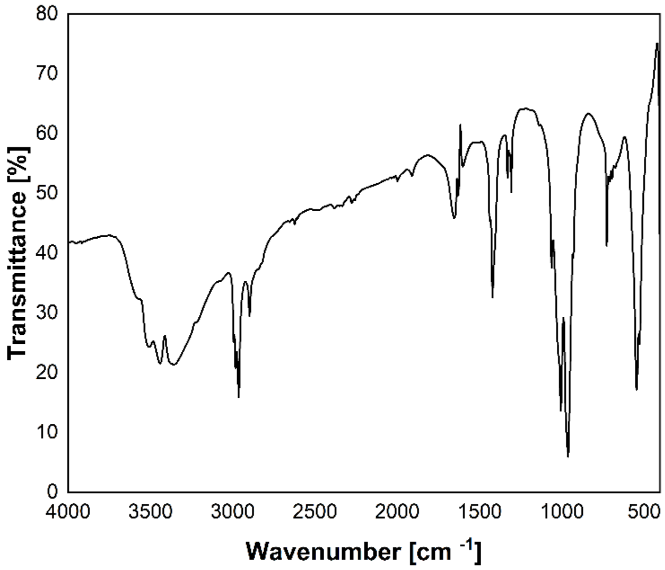

Figure S5. The results of FTIR spectroscopy for the products of oligomerization allyl alcohol obtained using [VO(TDA)(phen)] • 1.5 H_2O.

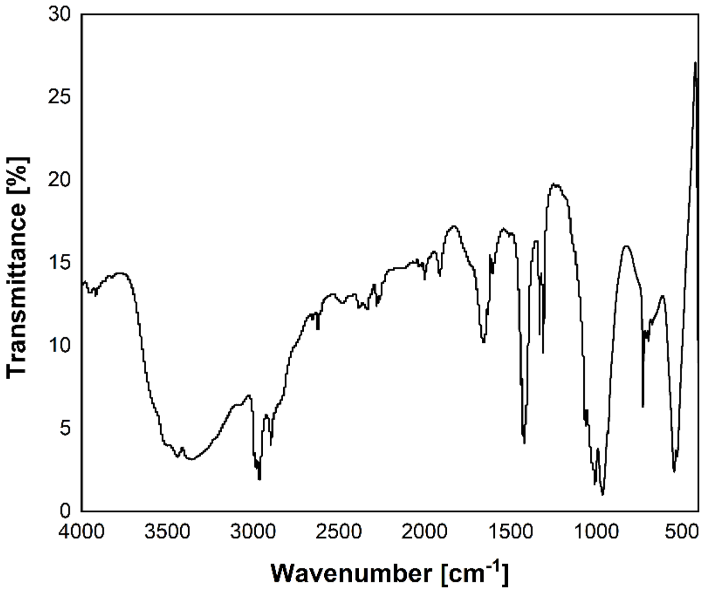

Figure S6. The results of FTIR spectroscopy for the products of oligomerization allyl alcohol obtained using [VOO(dipic)(2-phepyH)] • H_2O.

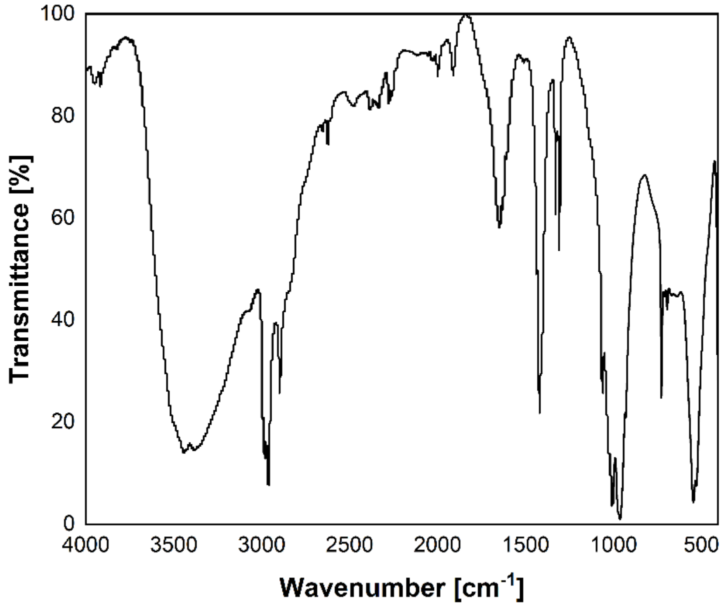

Figure S7. The results of FTIR spectroscopy for the products of oligomerization allyl alcohol obtained using [VO(dipic)(dmbipy)] • 2 H_2O.

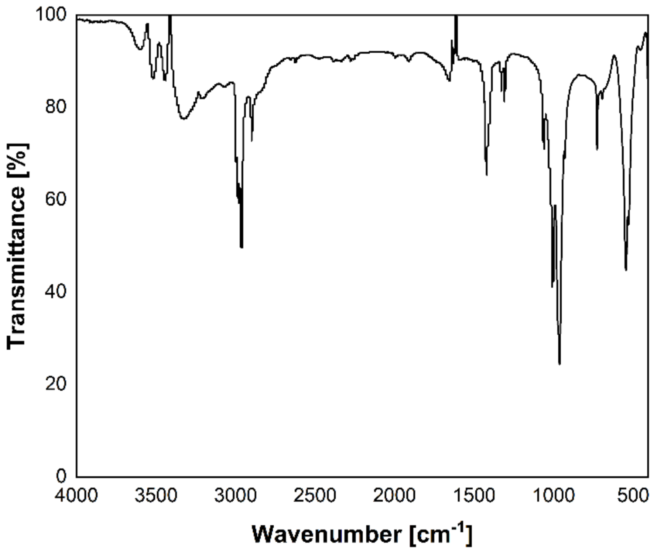

Figure S8. The results of FTIR spectroscopy for the products of oligomerization allyl alcohol obtained using [VO(ODA)bipy] • 2 H_2O.

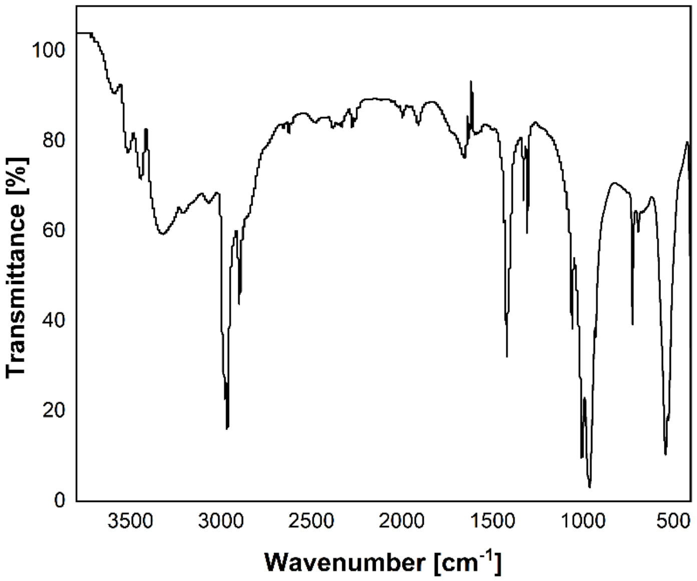

Figure S9. The results of FTIR spectroscopy for the products of oligomerization 2,3-dibromo-2-propen-1-ol obtained using [VO(TDA)(phen)] • 1.5 H_2O.

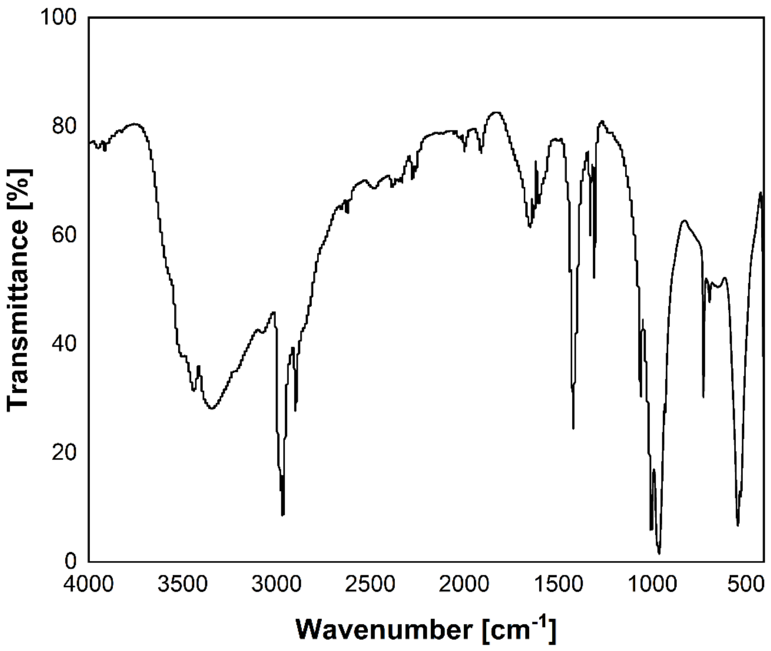

Figure S10. The results of FTIR spectroscopy for the products of oligomerization 2,3-dibromo-2-propen-1-ol obtained using [VOO(dipic)(2-phepyH)] • H_2O.

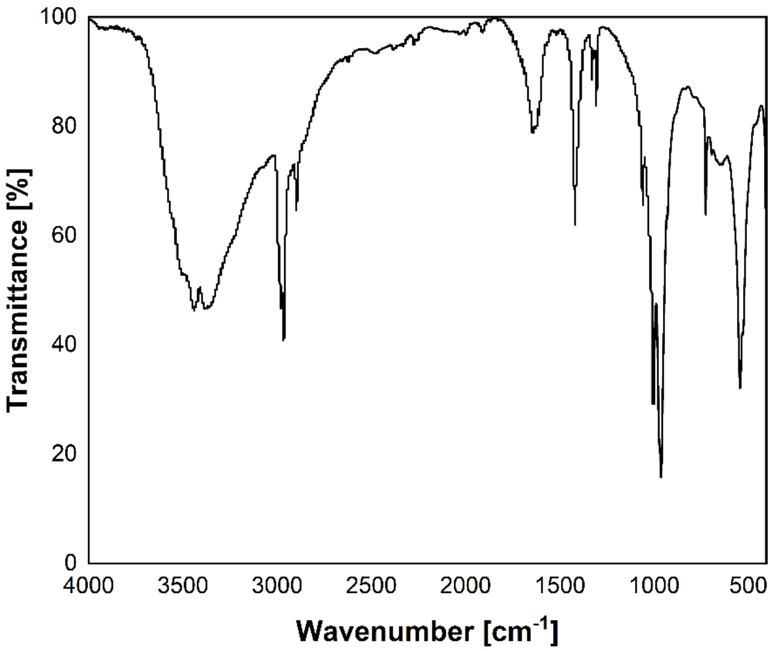

Figure S11. The results of FTIR spectroscopy for the products of oligomerization 2,3-dibromo-2-propen-1-ol obtained using [VO(dipic)(dmbipy)] • 2 H_2O.

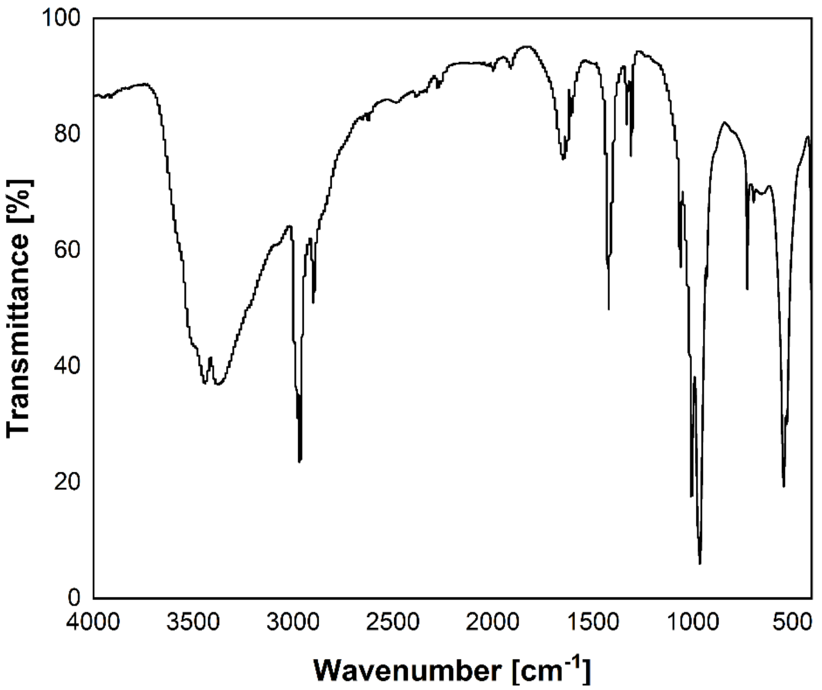

Figure S12. The results of FTIR spectroscopy for the products of oligomerization 2,3-dibromo-2-propen-1-ol obtained using [VO(ODA)bipy] • 2 H_2O.

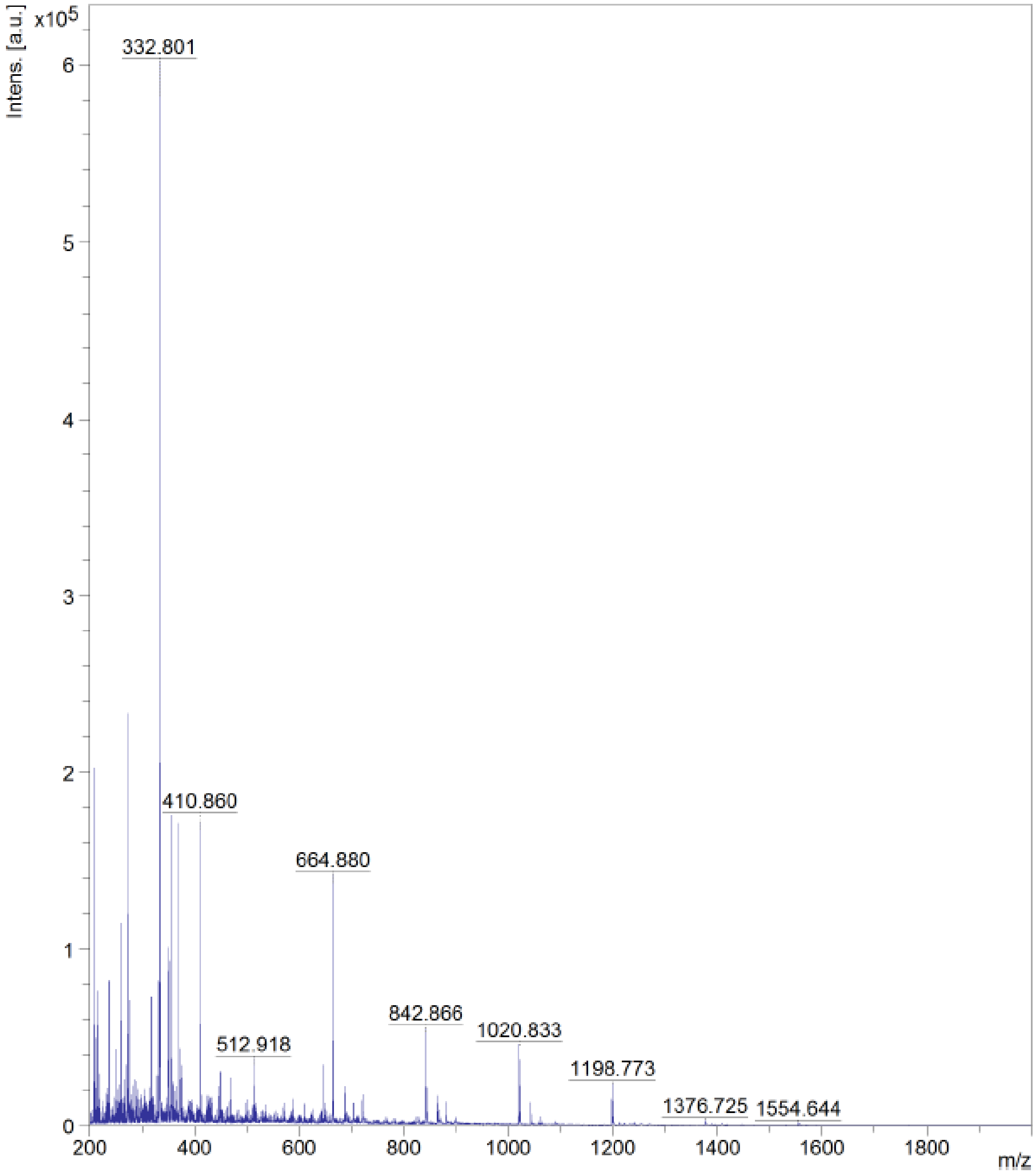

Figure S13. The MALDI-TOF-MS spectrum of the 3-buten-2-ol oligomer obtained using [VO(TDA)(phen)] • 1.5 H_2O.

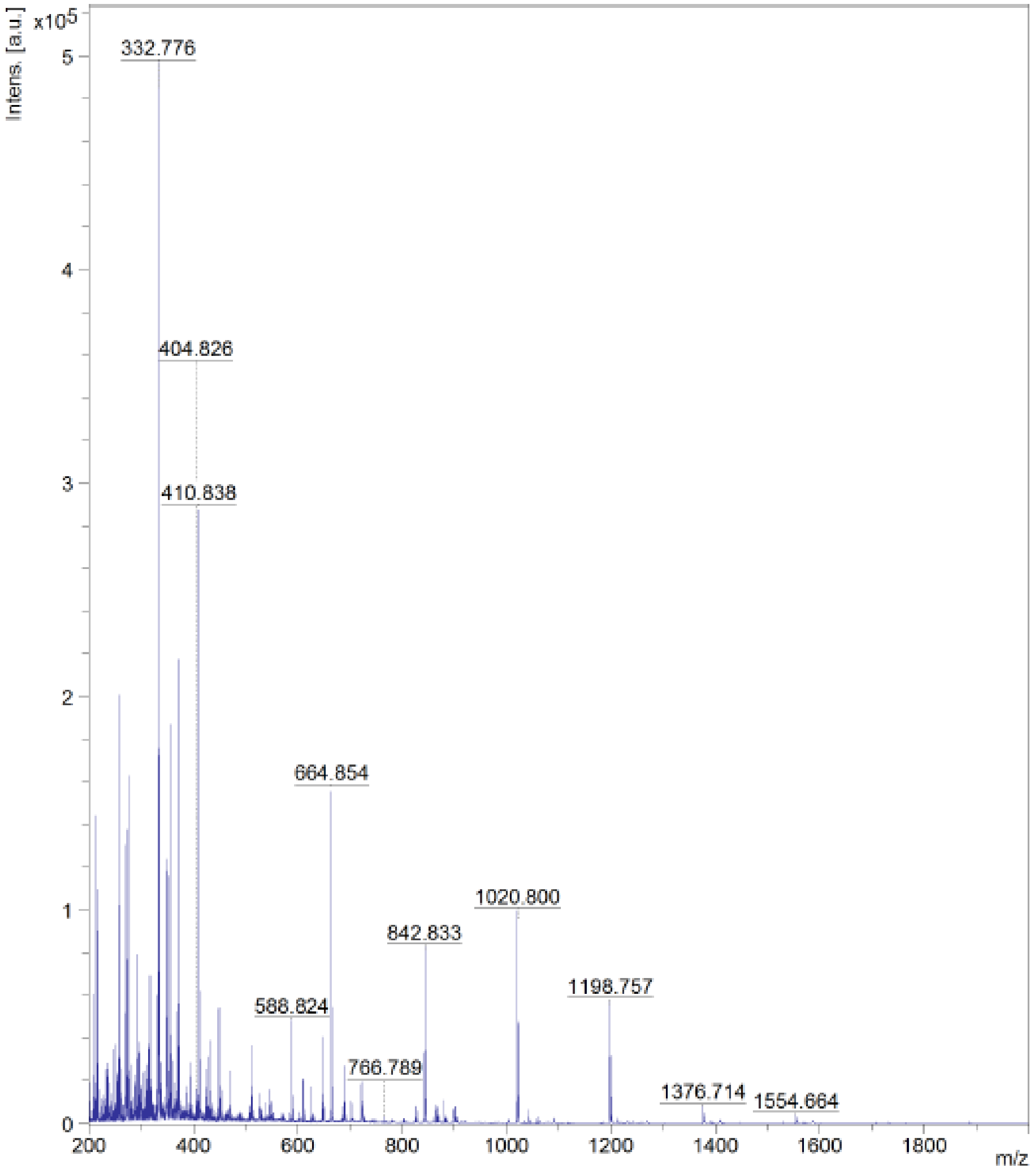

Figure S14. The MALDI-TOF-MS spectrum of the allyl alcohol oligomer obtained using [VO(TDA)(phen)] • 1.5 H_2O.

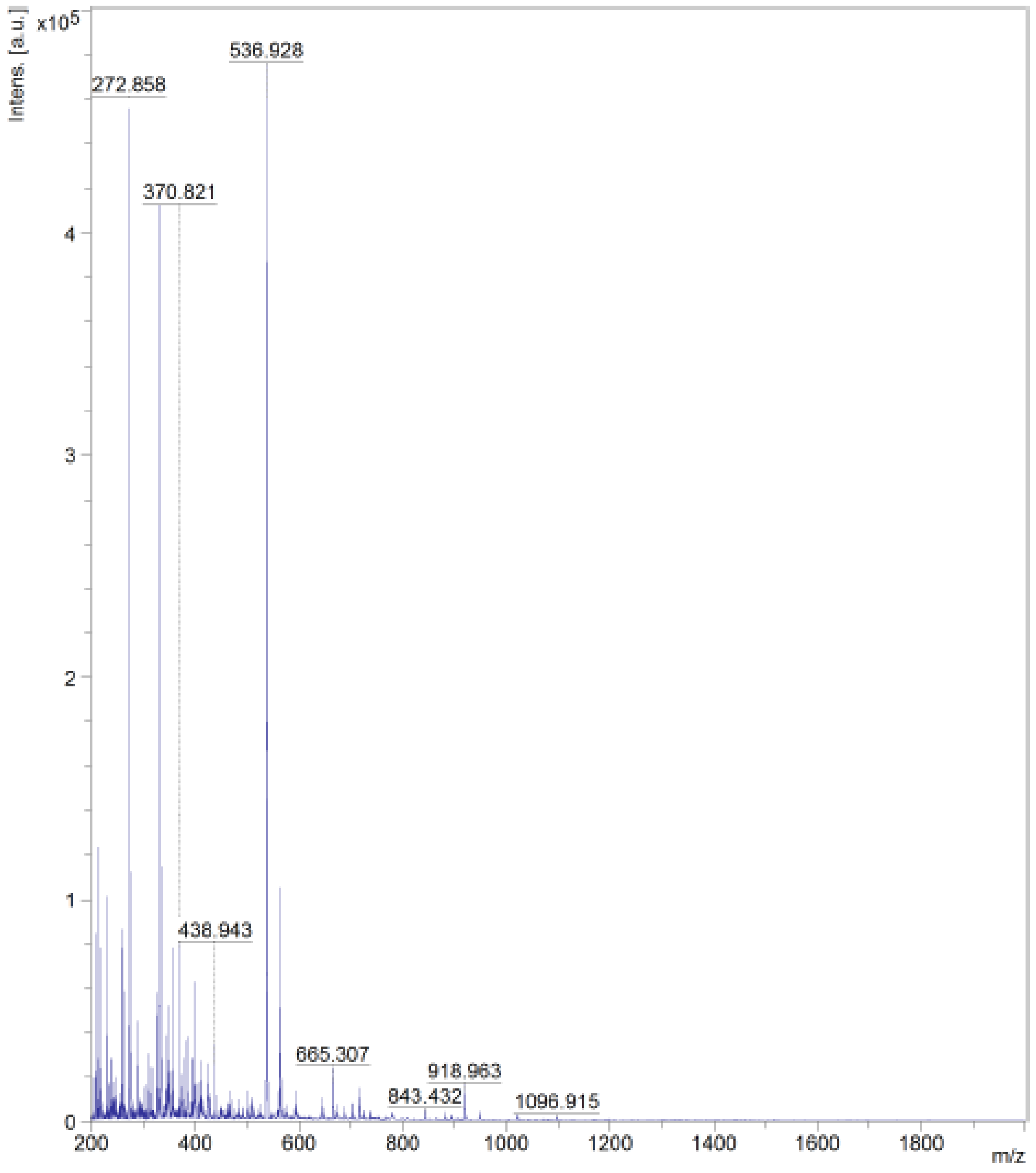

Figure S15. The MALDI-TOF-MS spectrum of the 2,3-dibromo-2-propen-1-ol oligomer obtained using [VO(TDA)(phen)] • 1.5 H_2O.

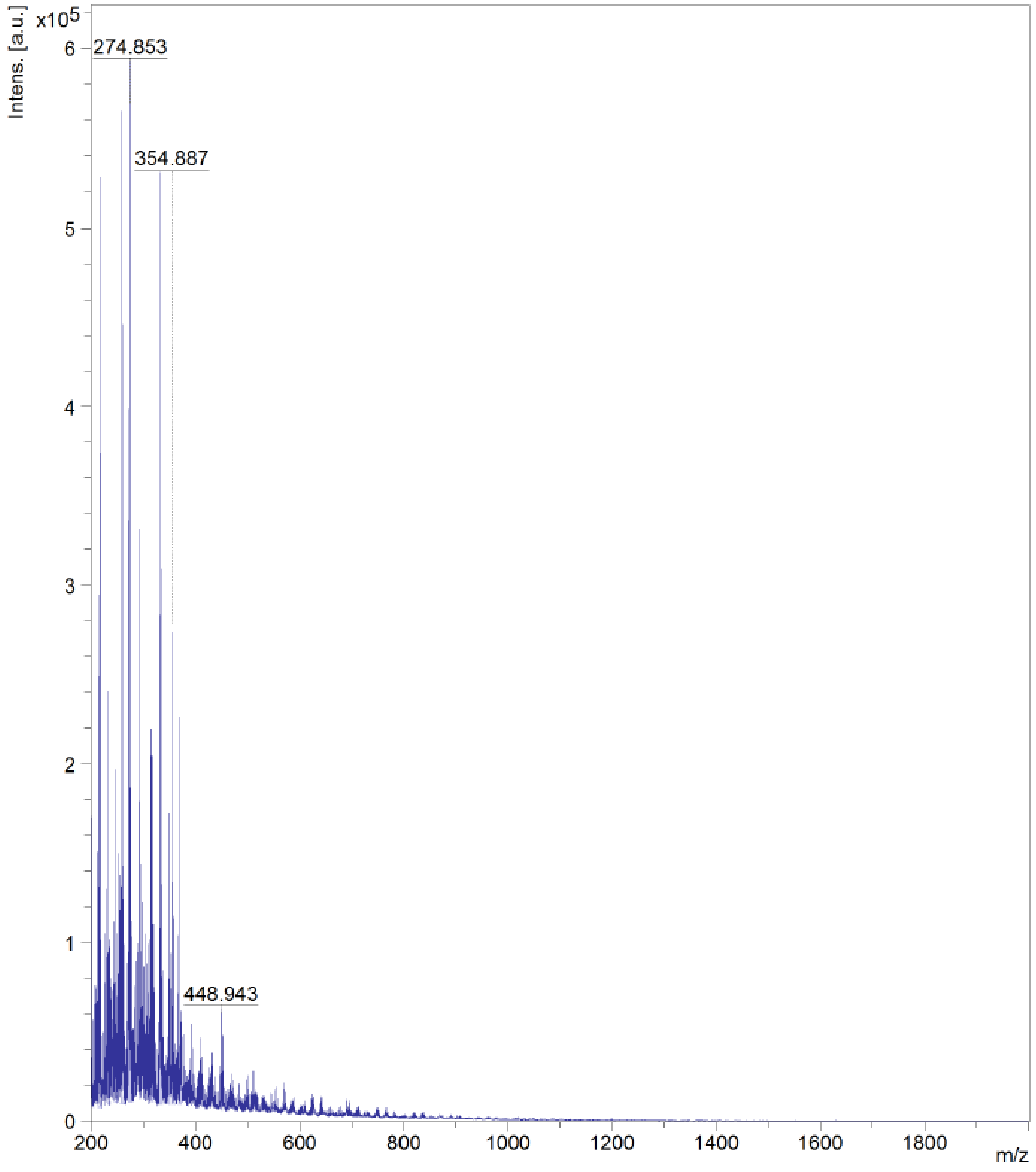

Figure S16. The MALDI-TOF-MS spectrum of the 3-buten-2-ol oligomer obtained using [VOO(dipic)(2-phepyH)] • H_2O.

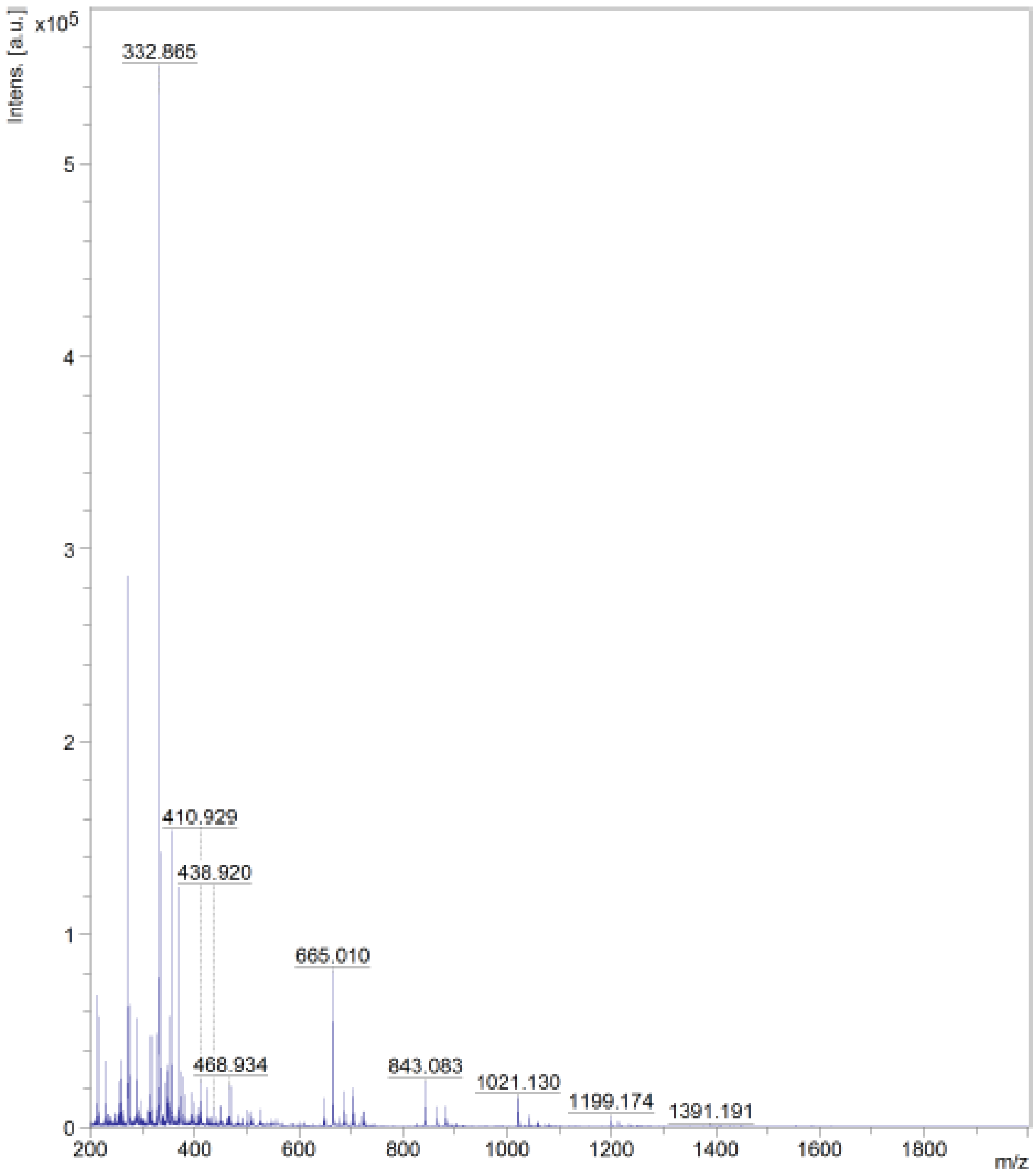

Figure S17. The MALDI-TOF-MS spectrum of the allyl alcohol oligomer obtained using [VOO(dipic)(2-phepyH)] • H_2O.

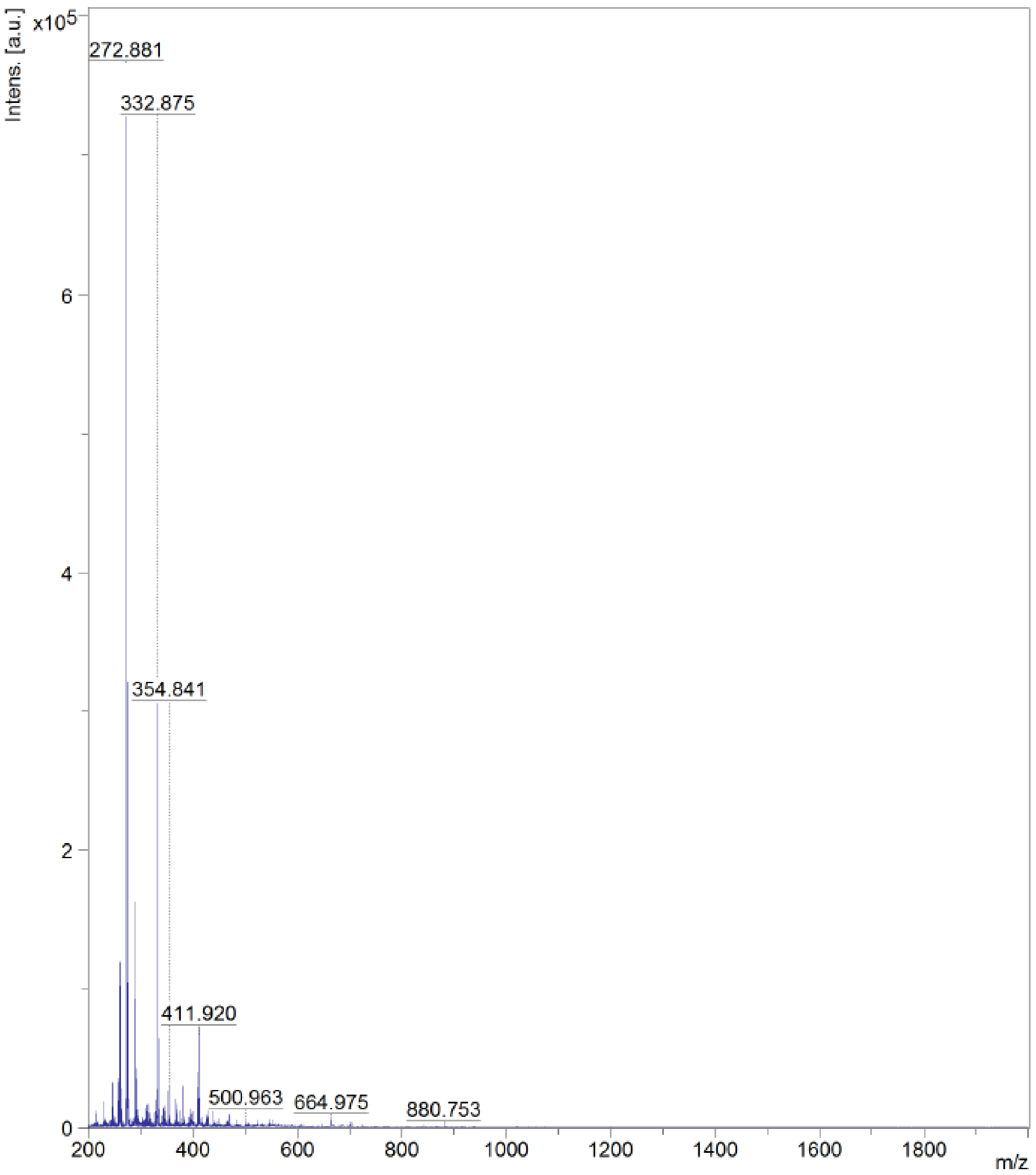

Figure S18. The MALDI-TOF-MS spectrum of the 2,3-dibromo-2-propen-1-ol oligomer obtained using [VOO(dipic)(2-phepyH)] • H_2O.

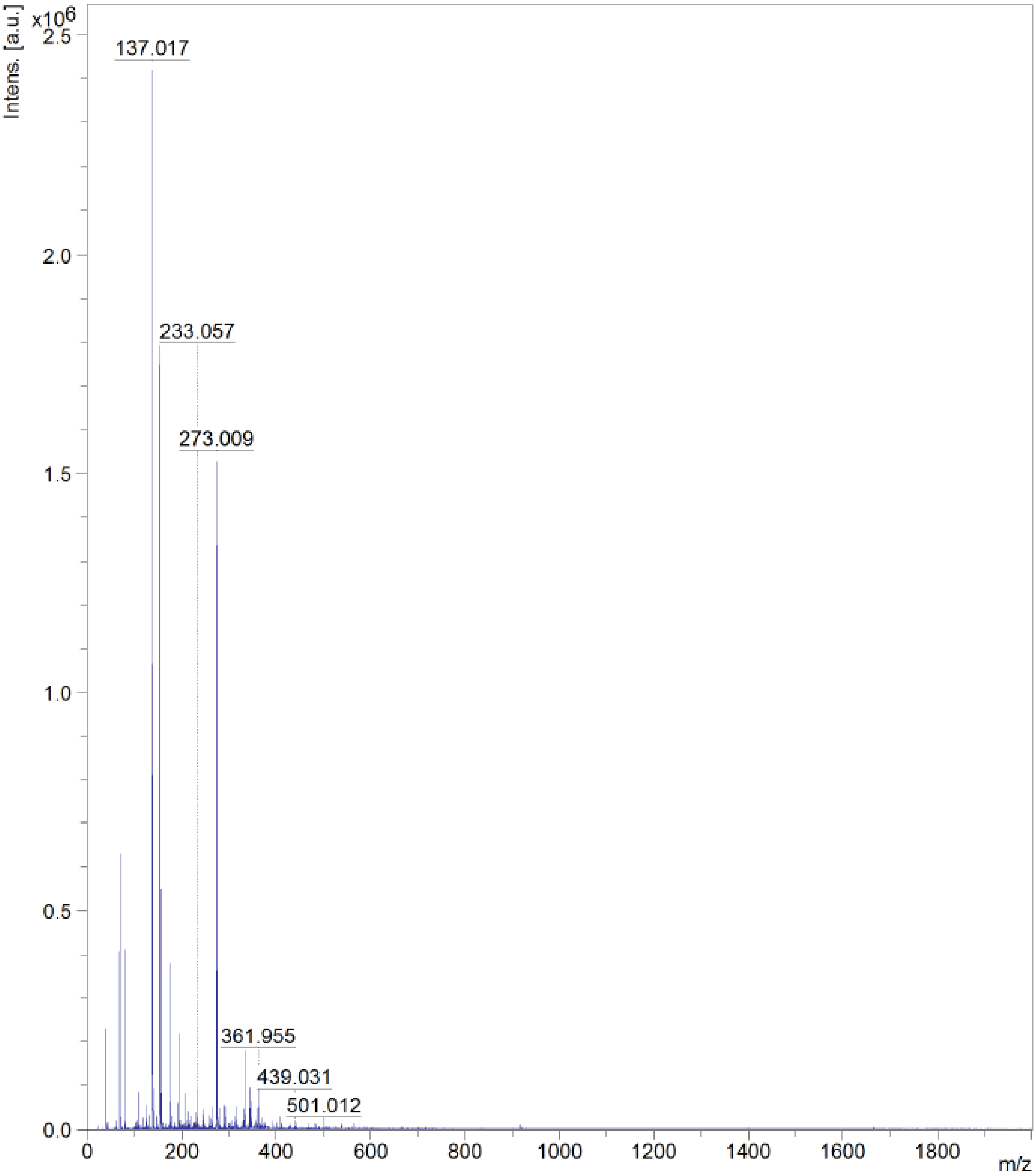

Figure S19. The MALDI-TOF-MS spectrum of the 3-buten-2-ol oligomer obtained using [VO(dipic)(dmbipy)] • 2 H_2O.

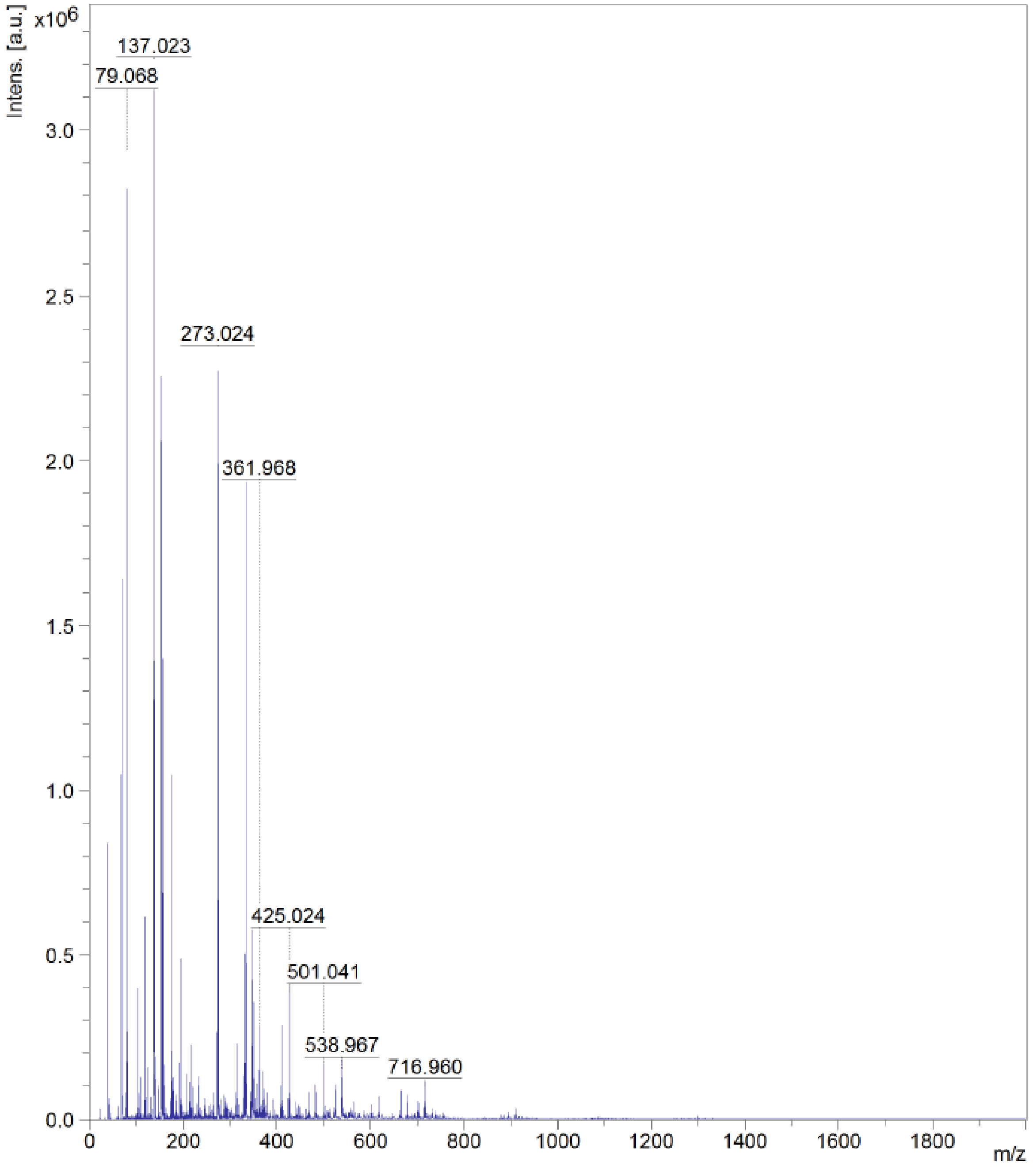

Figure S20. The MALDI-TOF-MS spectrum of the allyl alcohol oligomer obtained using [VO(dipic)(dmbipy)] • 2 H_2O.

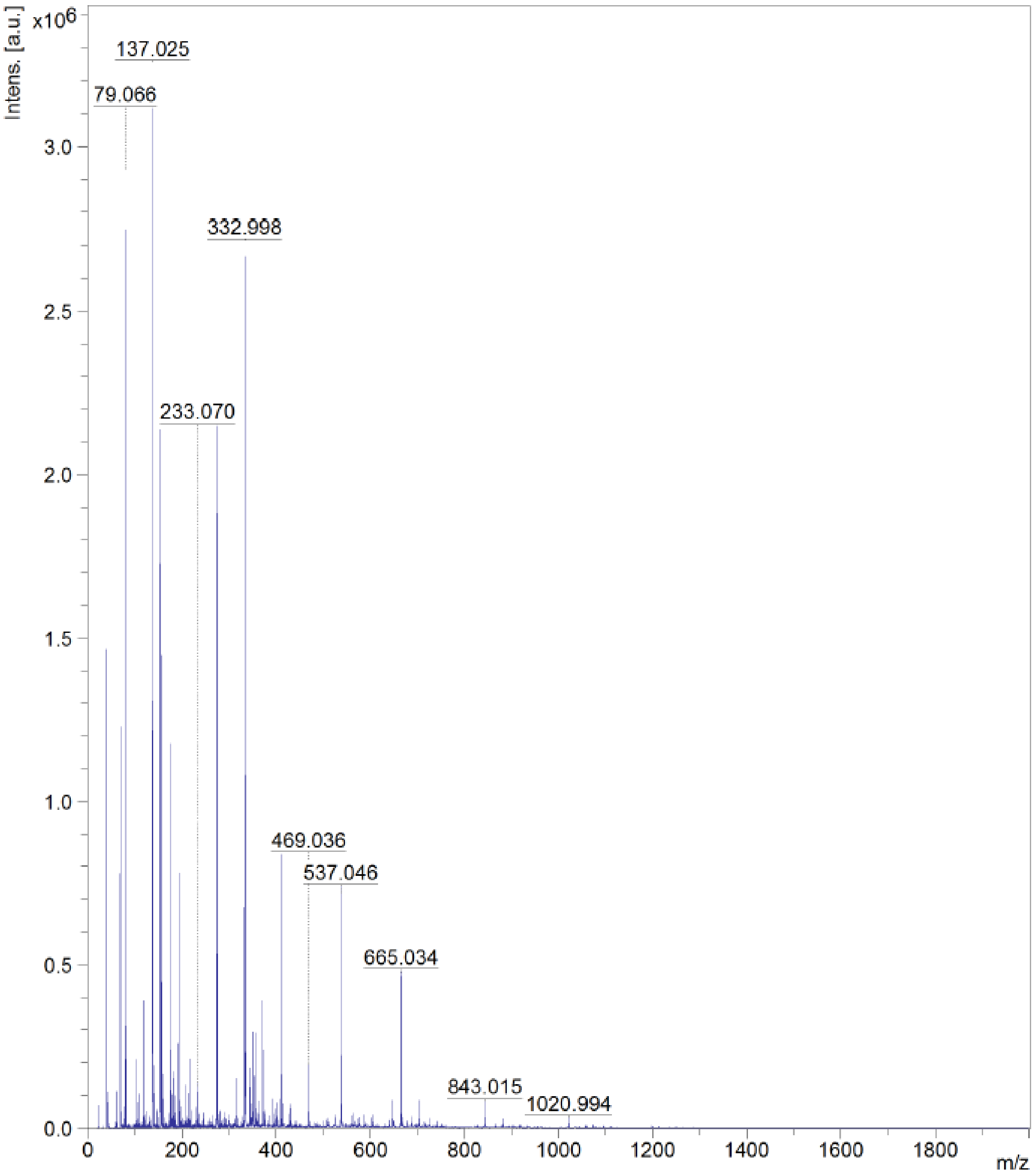

Figure S21. The MALDI-TOF-MS spectrum of the 2,3-dibromo-2-propen-1-ol oligomer obtained using [VO(dipic)(dmbipy)] • 2 H_2O.

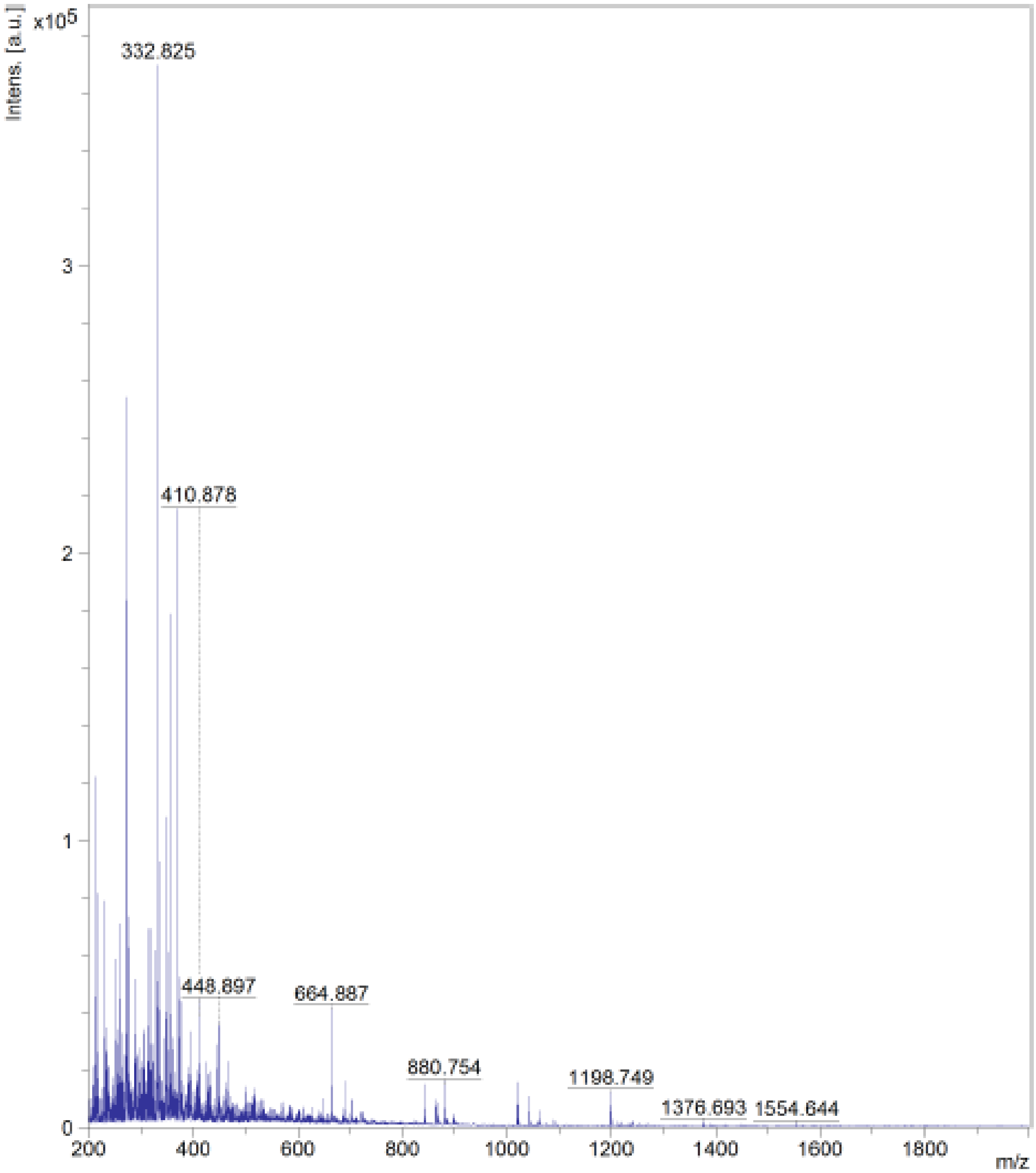

Figure S22. The MALDI-TOF-MS spectrum of the 3-buten-2-ol oligomer obtained using [VO(ODA)bipy] • 2 H_2O.

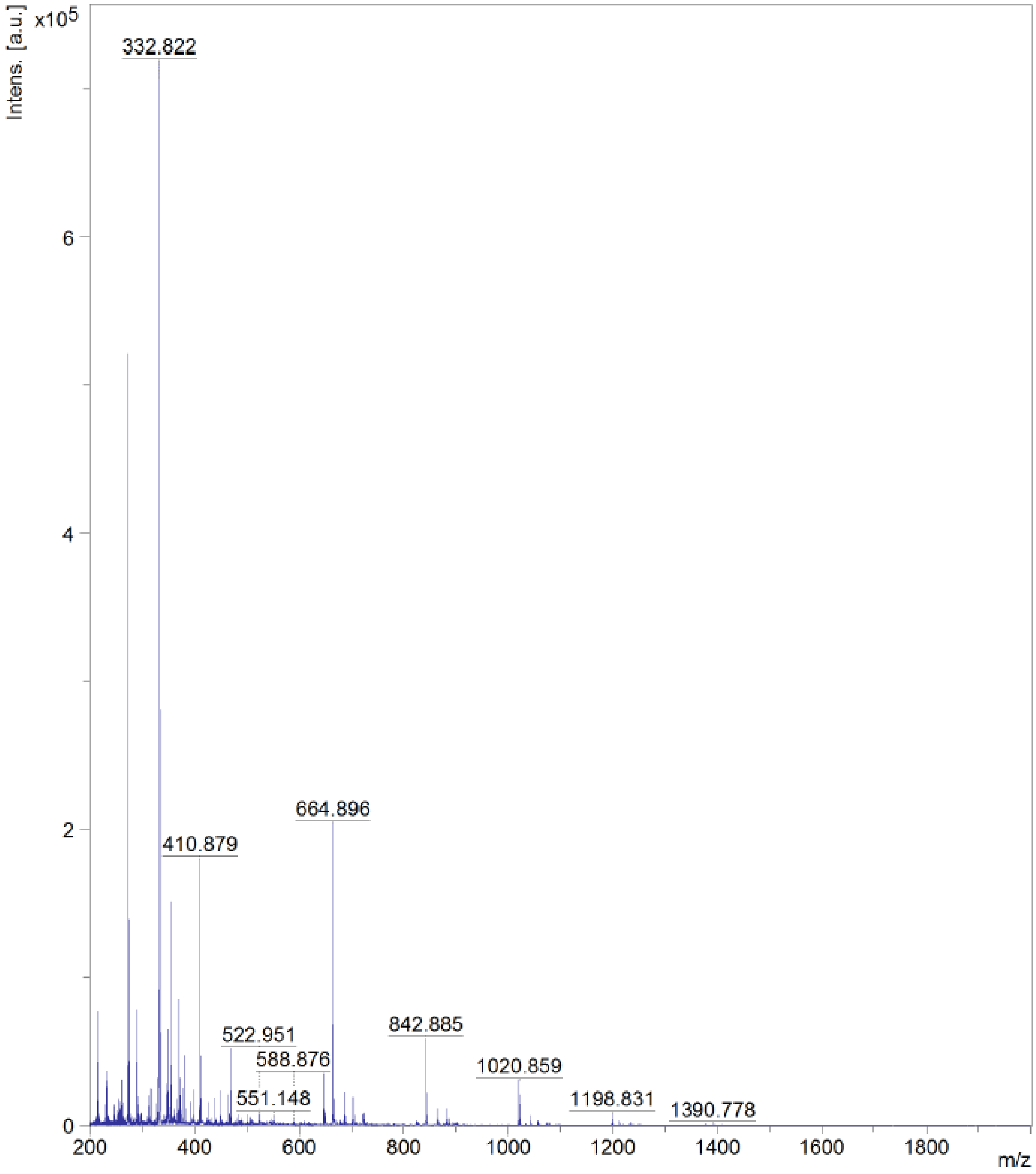

Figure S23. The MALDI-TOF-MS spectrum of the allyl alcohol oligomer obtained using [VO(ODA)bipy] • 2 H_2O.

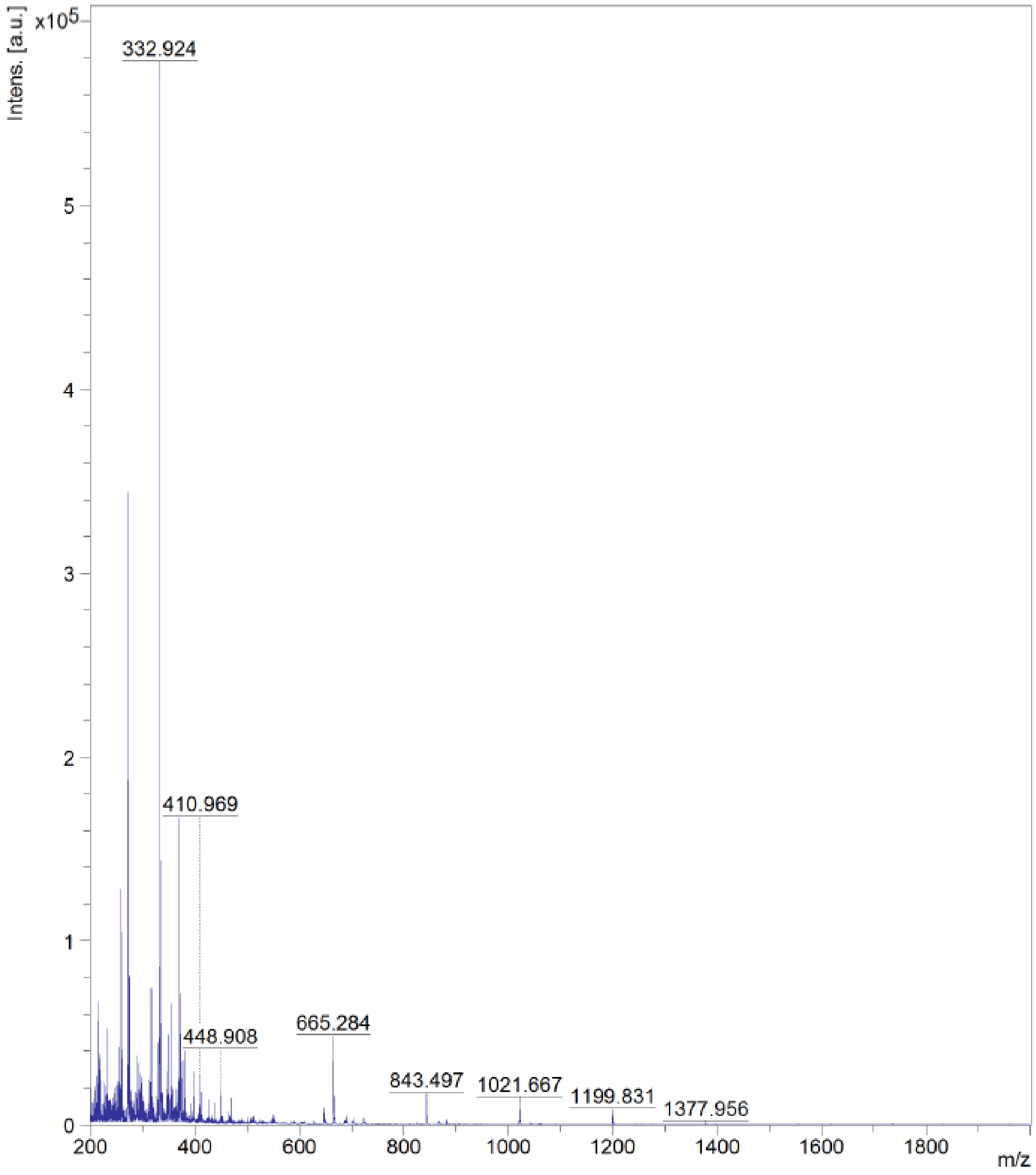

Figure S24. The MALDI-TOF-MS spectrum of the 2,3-dibromo-2-propen-1-ol oligomer obtained using [VO(ODA)bipy] • 2 H_2O.

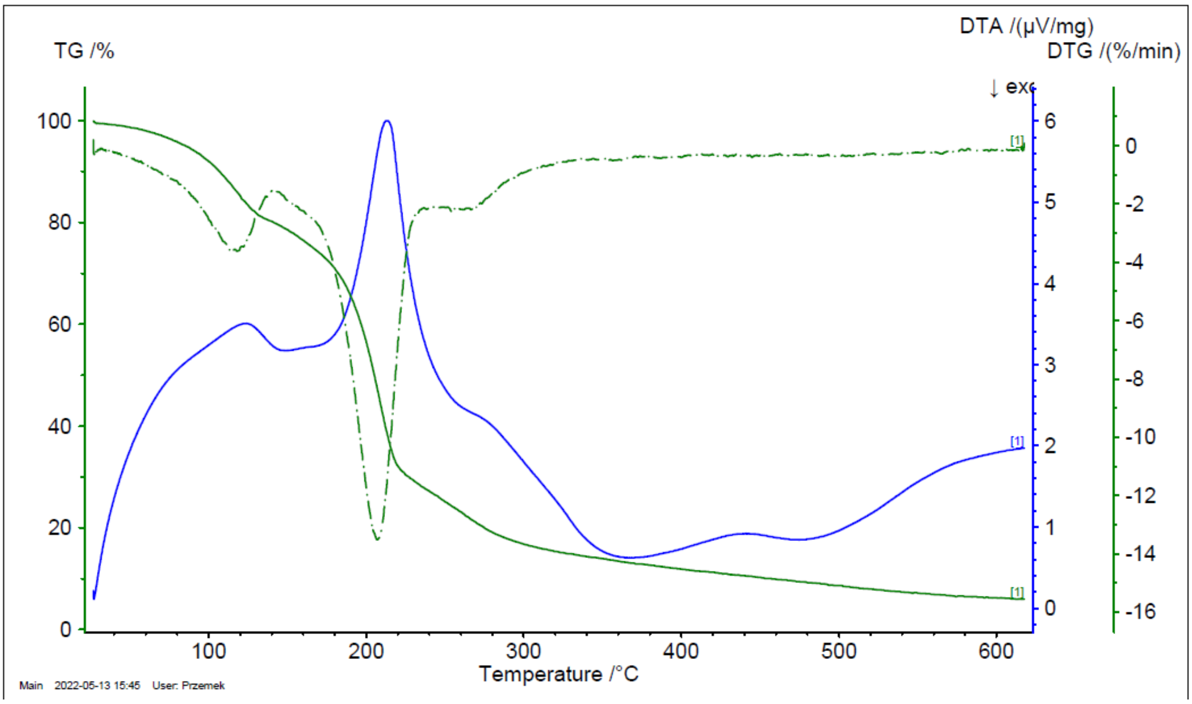

Figure S25. The TG spectrum of the 3-buten-2-ol oligomer obtained using [VO(TDA)phen] • 1.5 H_2O.

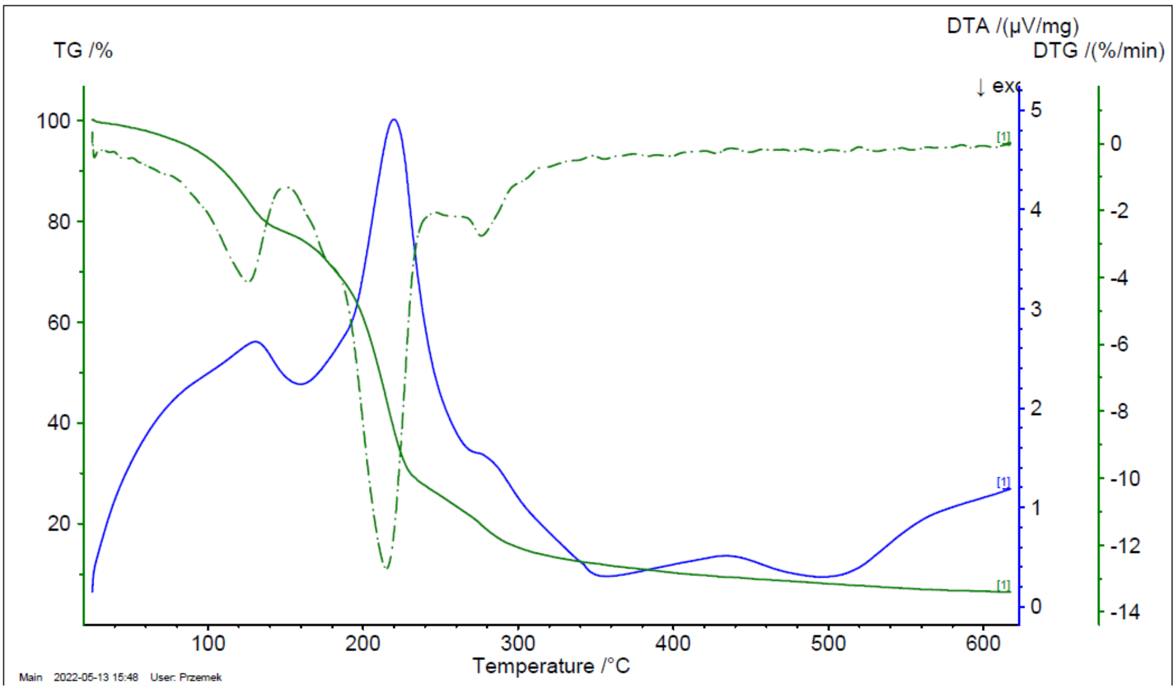

Figure S26. The TG spectrum of the allyl alcohol oligomer obtained using [VO(TDA)phen] • 1.5 H_2O.

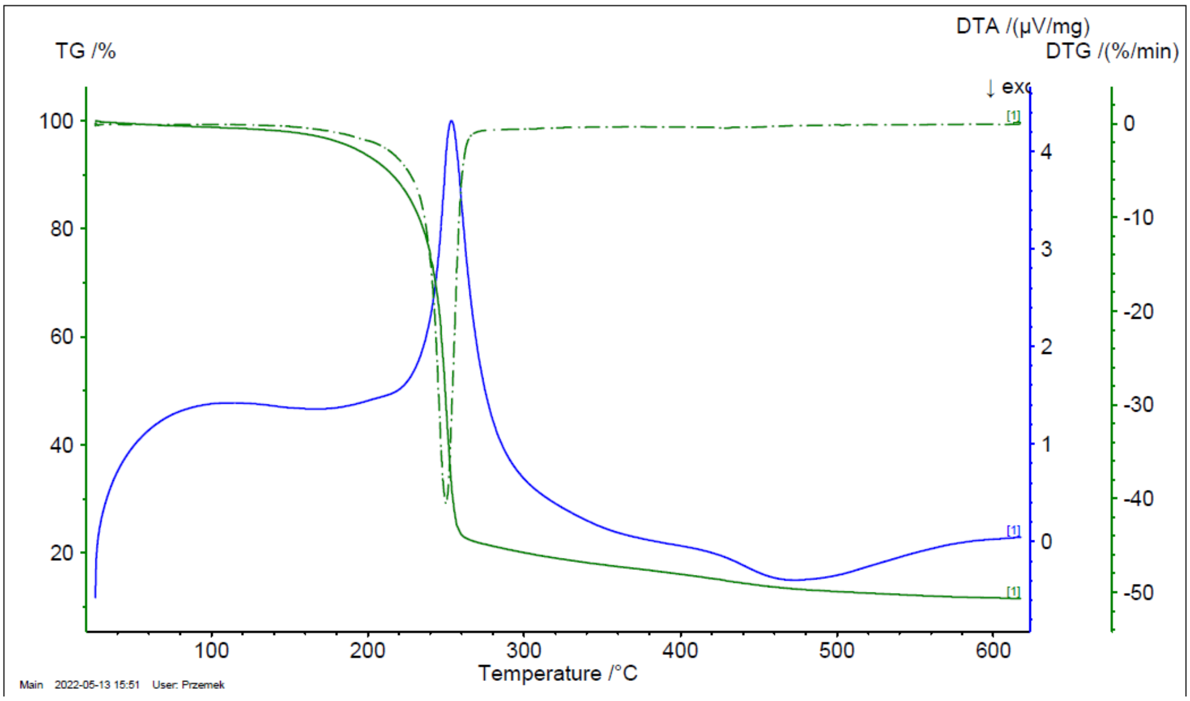

Figure S27. The TG spectrum of the 2,3-dibromo-2-propen-1-ol oligomer obtained using [VO(TDA)phen] • 1.5 H_2O.

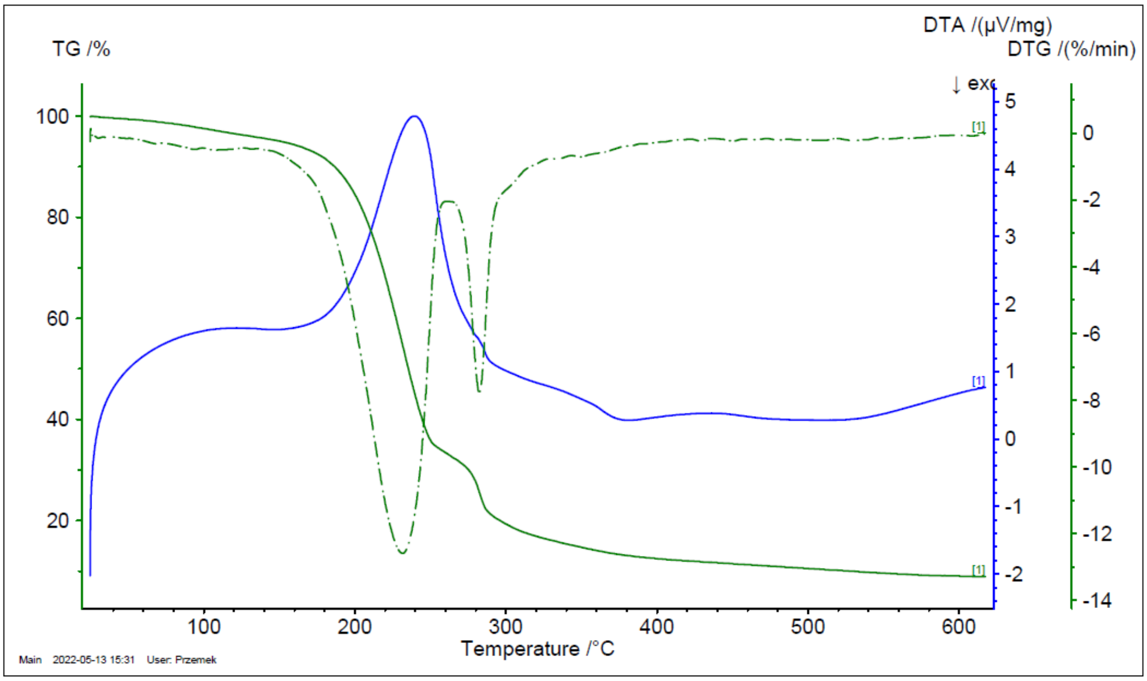

Figure S28. The TG spectrum of the 3-buten-2-ol oligomer obtained using [VOO(dipic)(2-phepyH)] • H_2O.

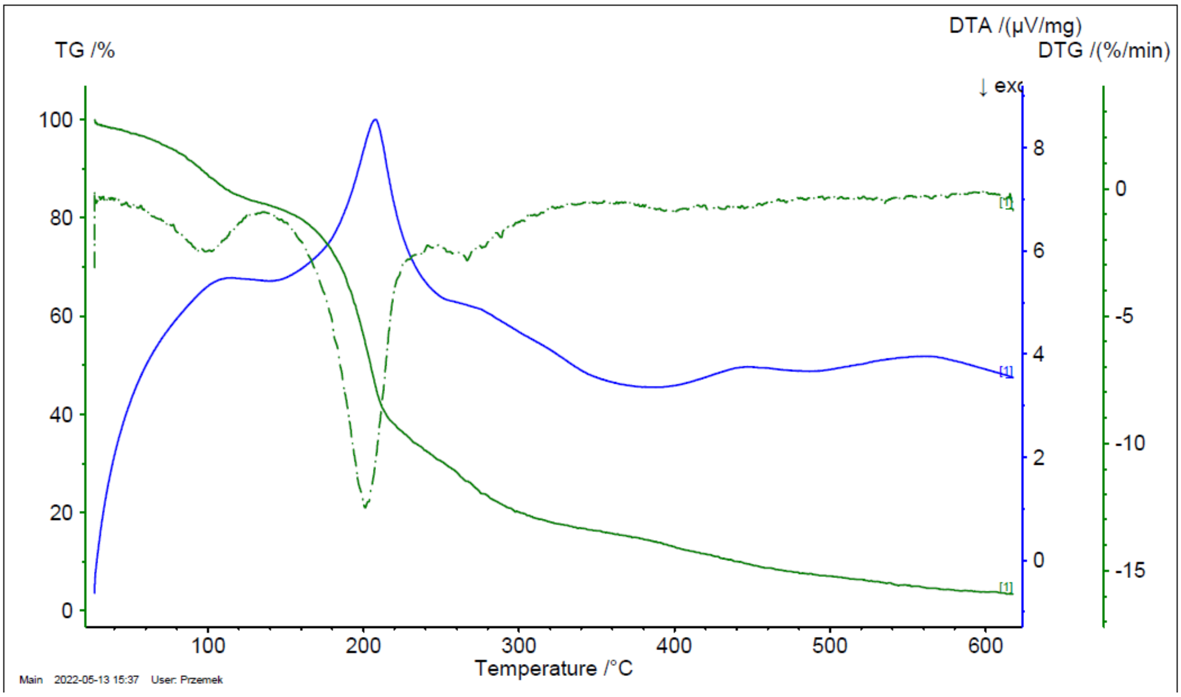

Figure S29. The TG spectrum of the allyl alcohol oligomer obtained using [VOO(dipic)(2-phepyH)] • H_2O.

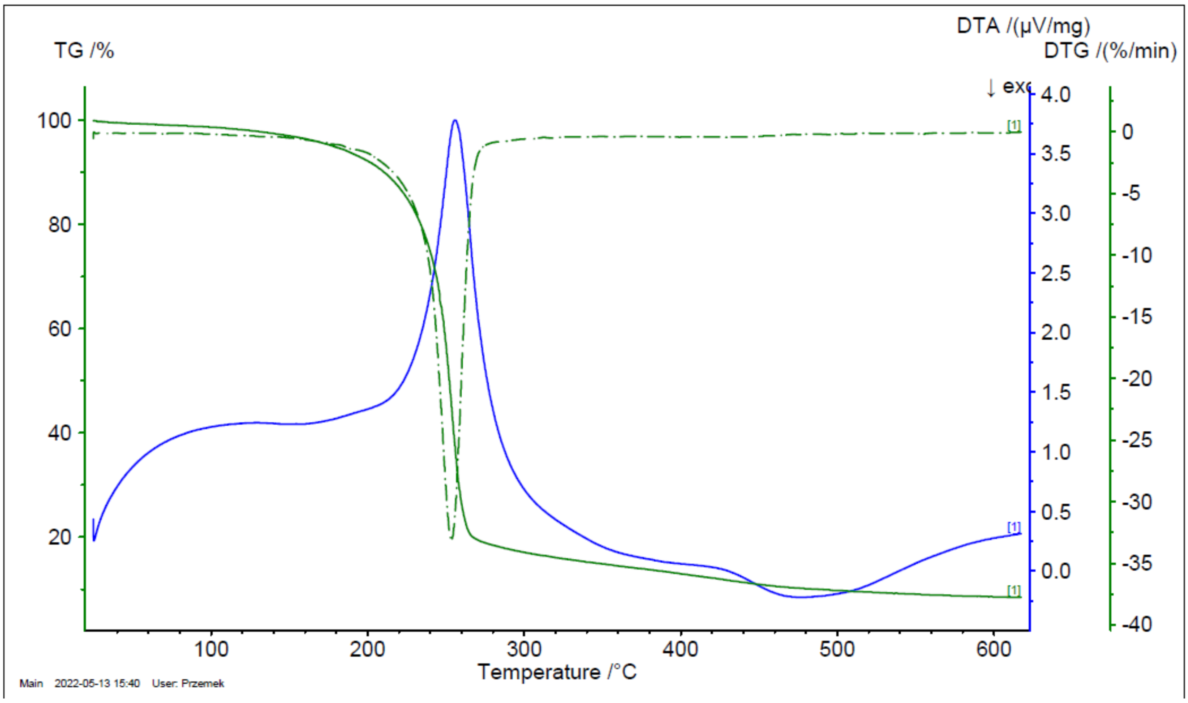

Figure S30 The TG spectrum of the 2,3-dibromo-2-propen-1-ol oligomer obtained using [VOO(dipic)(2-phepyH)] • H_2O.

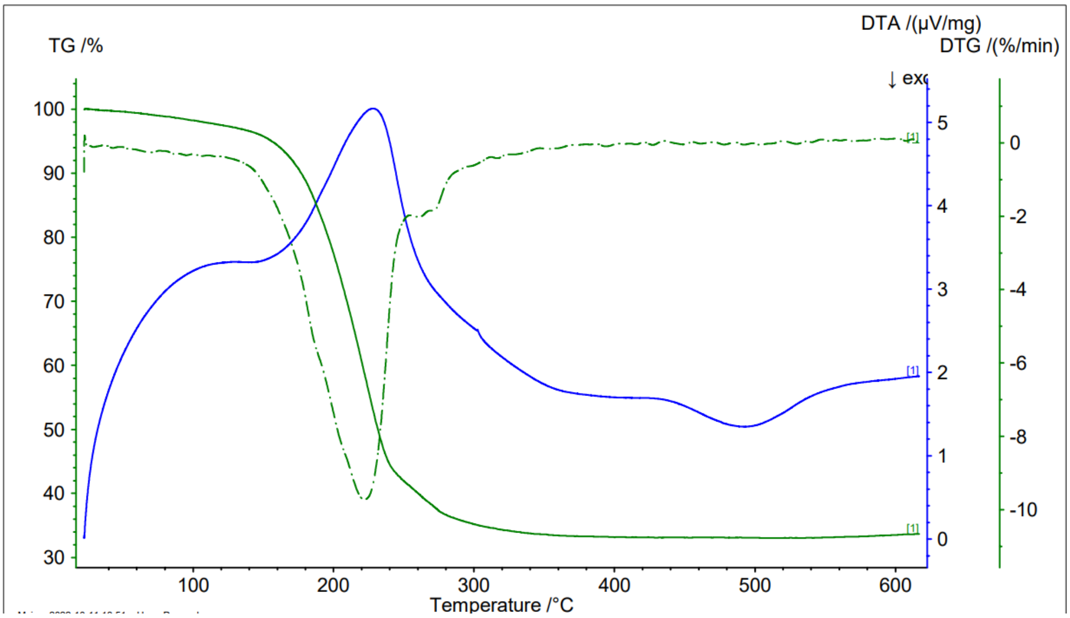

Figure S31. The TG spectrum of the 3-buten-2-ol oligomer obtained using [VO(dipic)(dmbipy)] • 2 H_2O

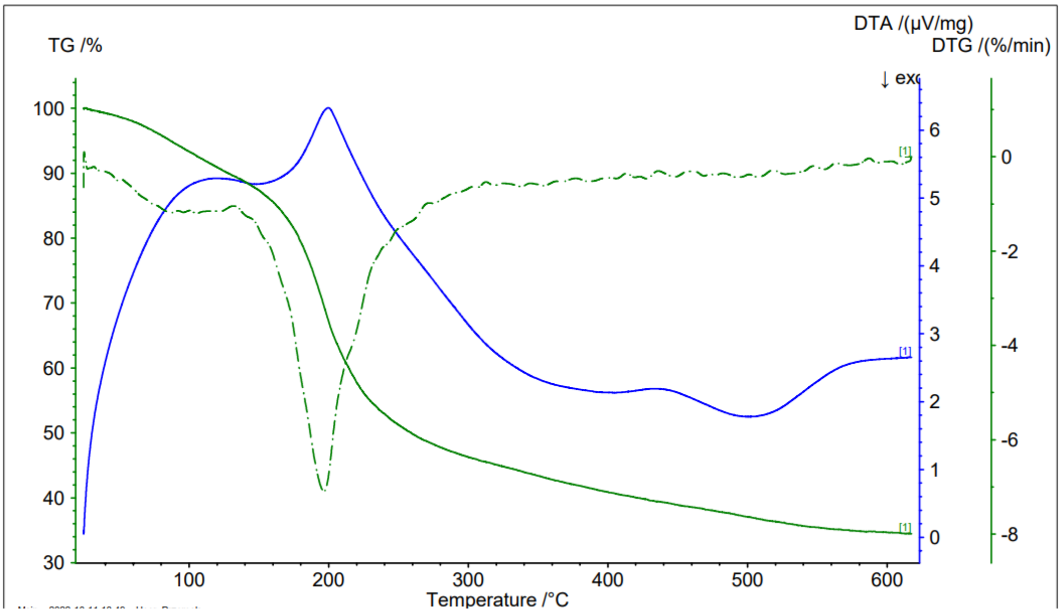

Figure S32. The TG spectrum of the allyl alcohol oligomer obtained using [VO(dipic)(dmbipy)] • 2 H_2O

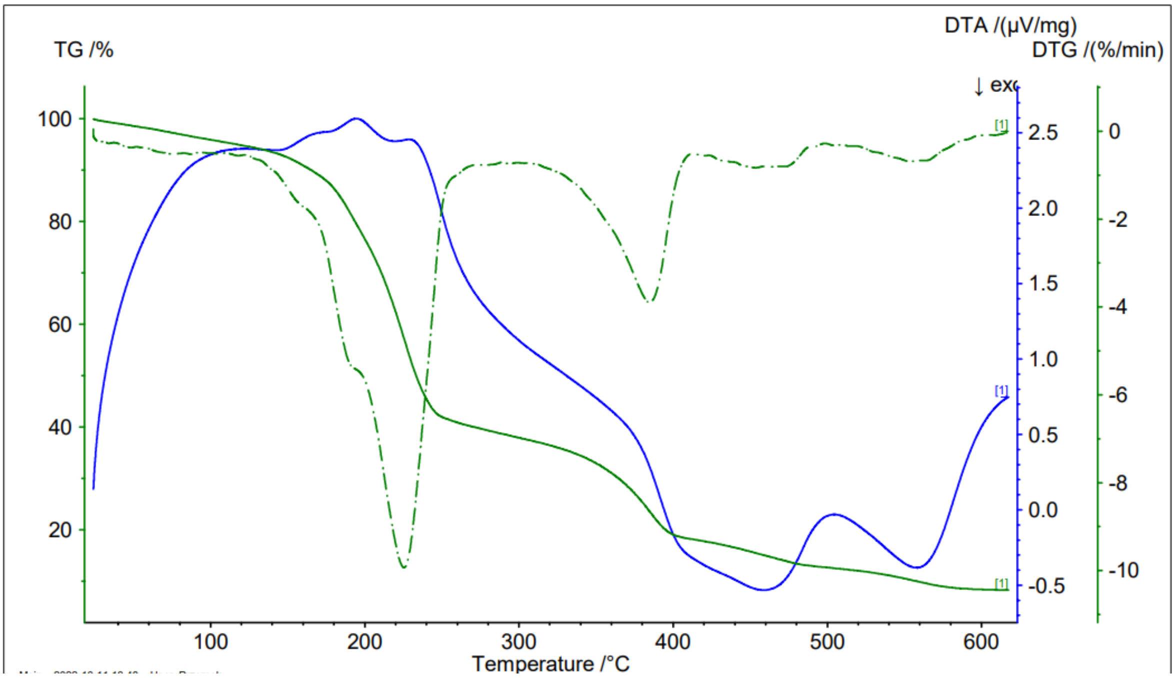

Figure S33. The TG spectrum of the 2,3-dibro-2-propen-1-ol oligomer obtained using [VO(dipic)(dmbipy)] • 2 H_2O

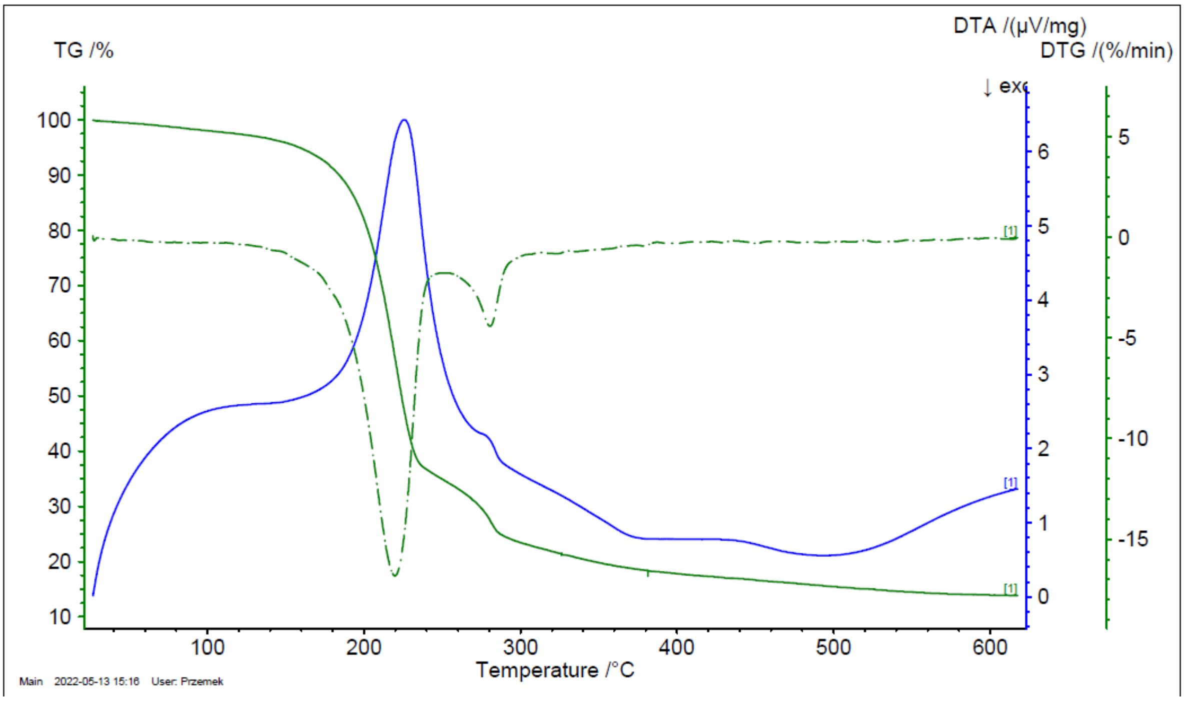

Figure S34. The TG spectrum of the 3-buten-2-ol oligomer obtained using [VO(ODA)bipy] • 2 H_2O

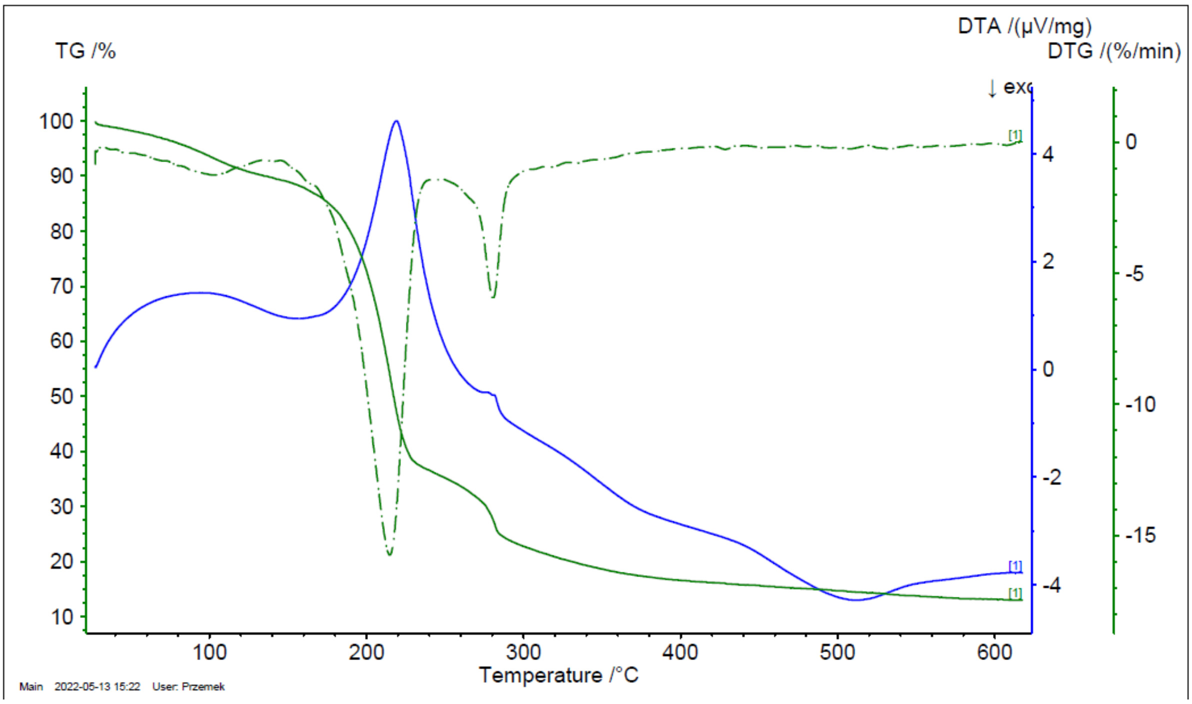

Figure S35. The TG spectrum of the allyl alcohol oligomer obtained using [VO(ODA)bipy] • 2 H_2O

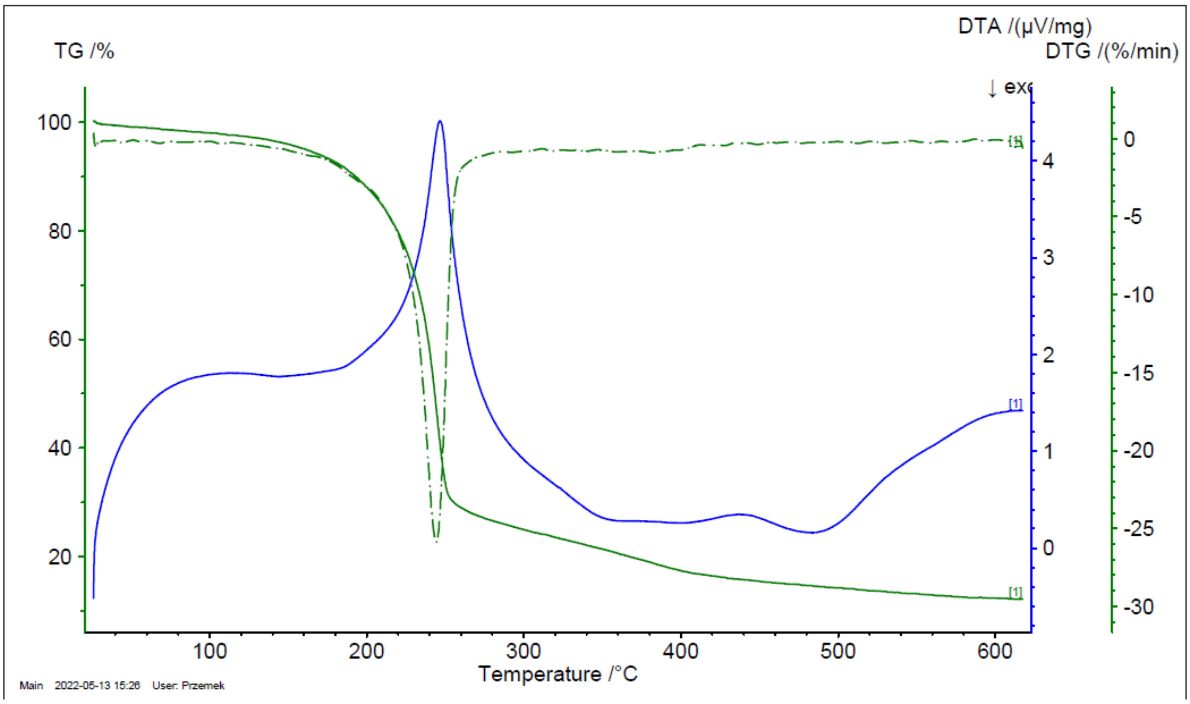

Figure S36. The TG spectrum of the 2,3-dibromo-2-propen-1-ol oligomer obtained using [VO(ODA)bipy] • 2 H_2O

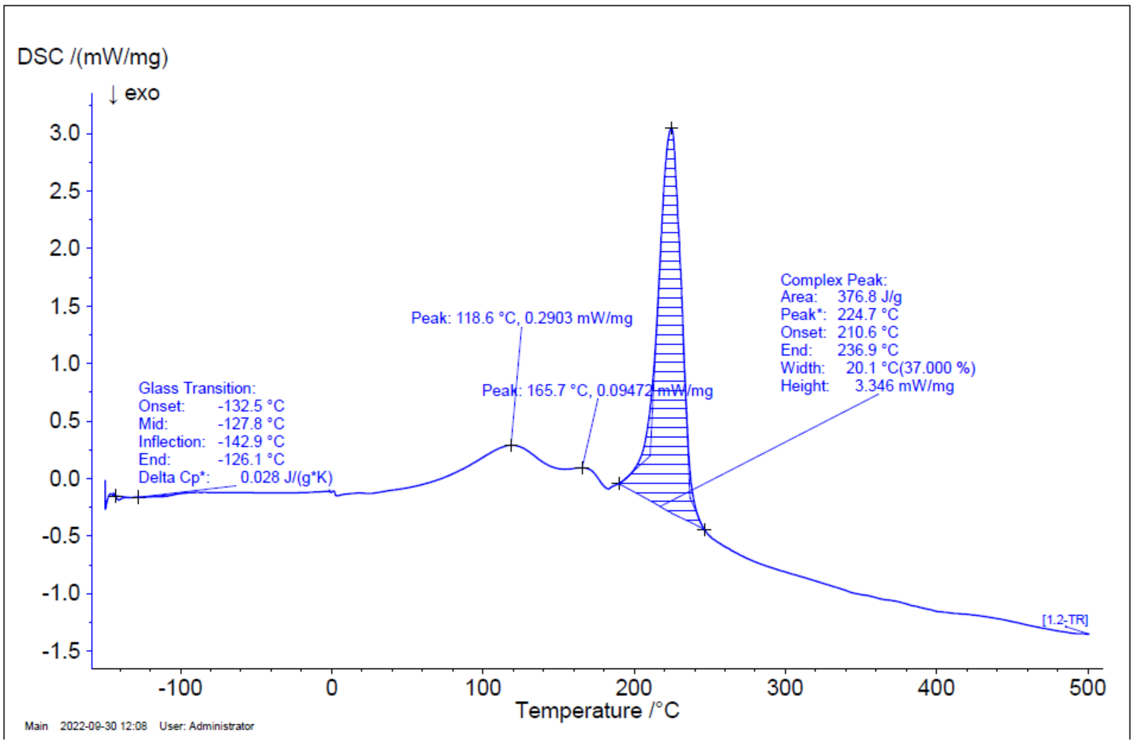

Figure S37. The DSC spectrum of the 3-buten-2-ol oligomer obtained using [VO(TDA)(phen)] • 1.5 H_2O.

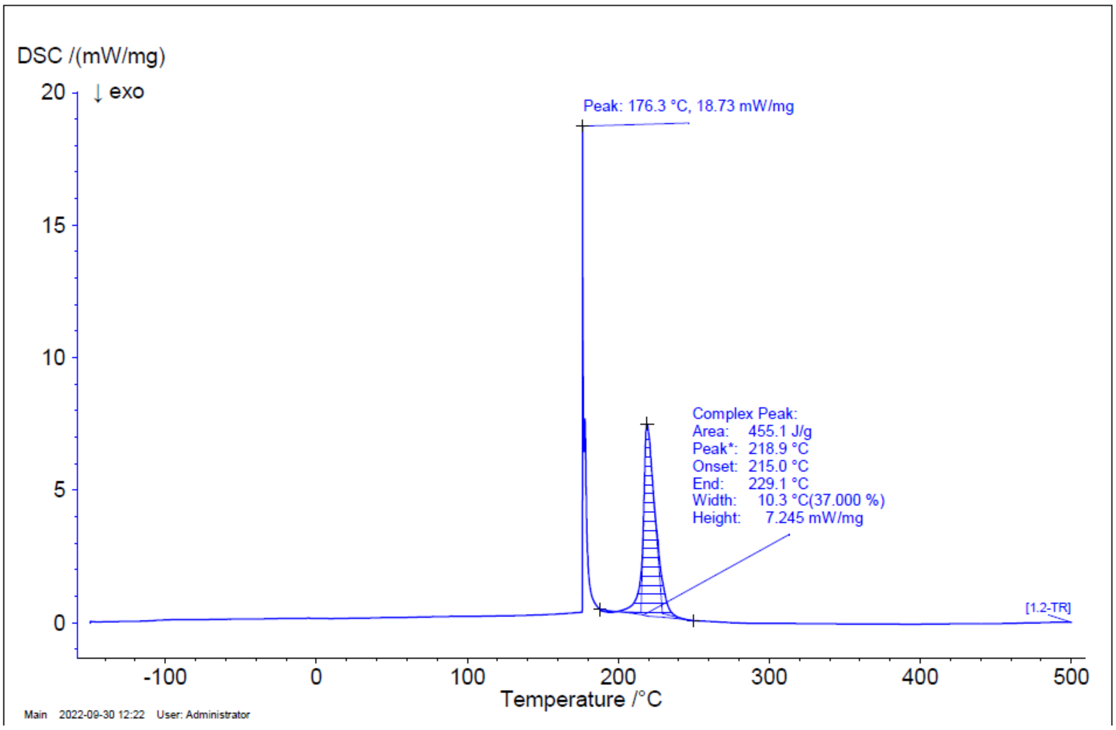

Figure S38. The DSC spectrum of the allyl alcohol oligomer obtained using [VO(TDA)(phen)] • 1.5 H_2O.

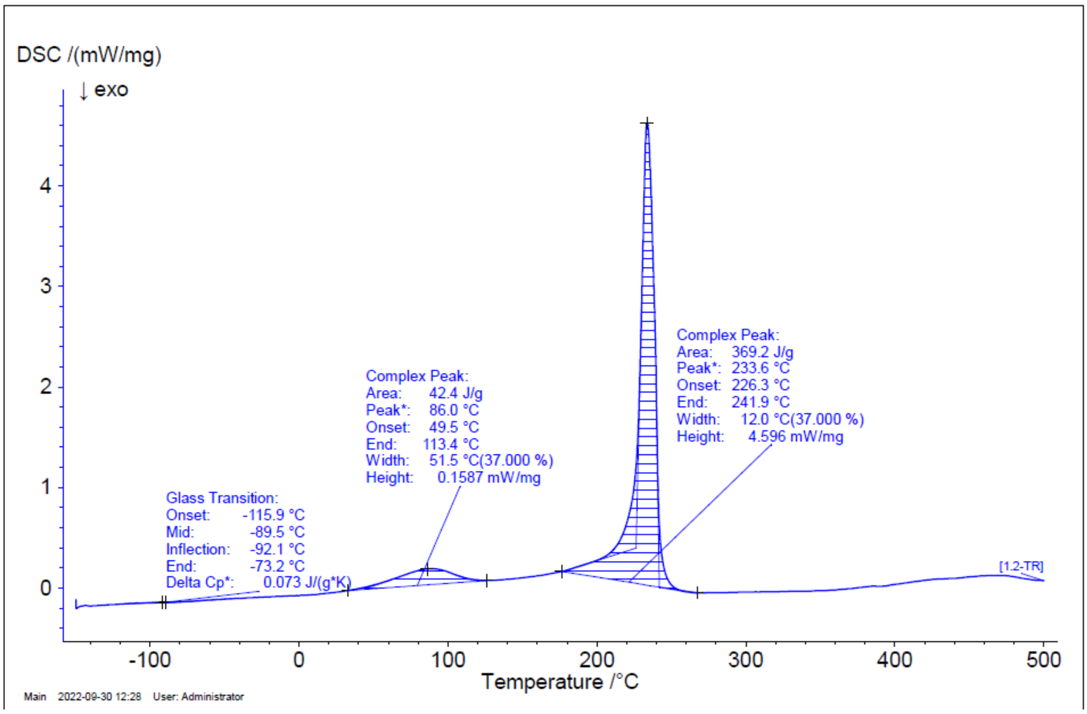

Figure S39. The DSC spectrum of the 2,3-dibromo-2-propen-1-ol oligomer obtained using [VO(TDA)(phen)] • 1.5 H_2O.

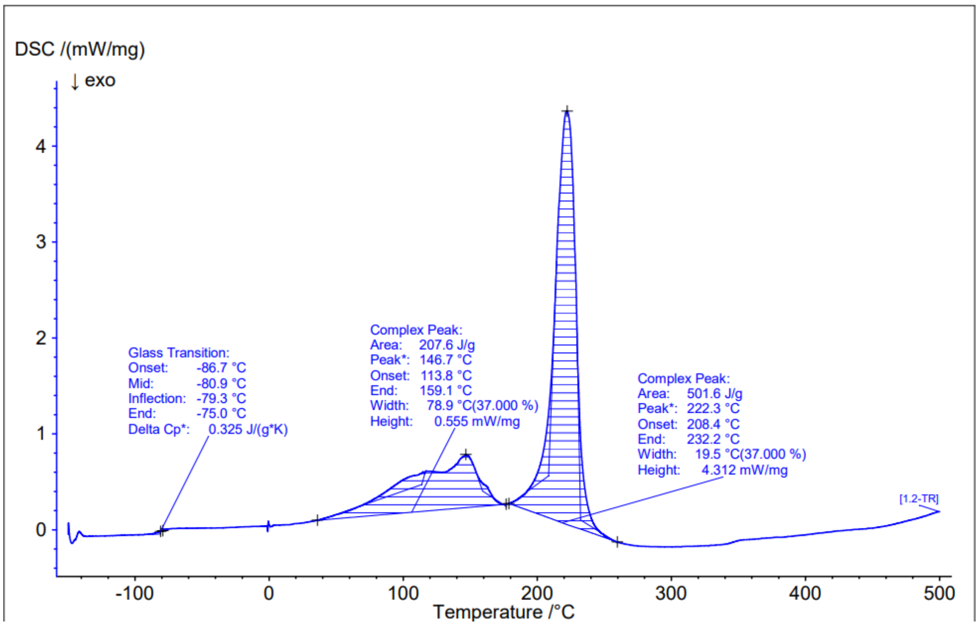

Figure S40. The DSC spectrum of the 3-buten-2-ol oligomer obtained using [VOO(dipic)(2-phepyH)] • H_2O.

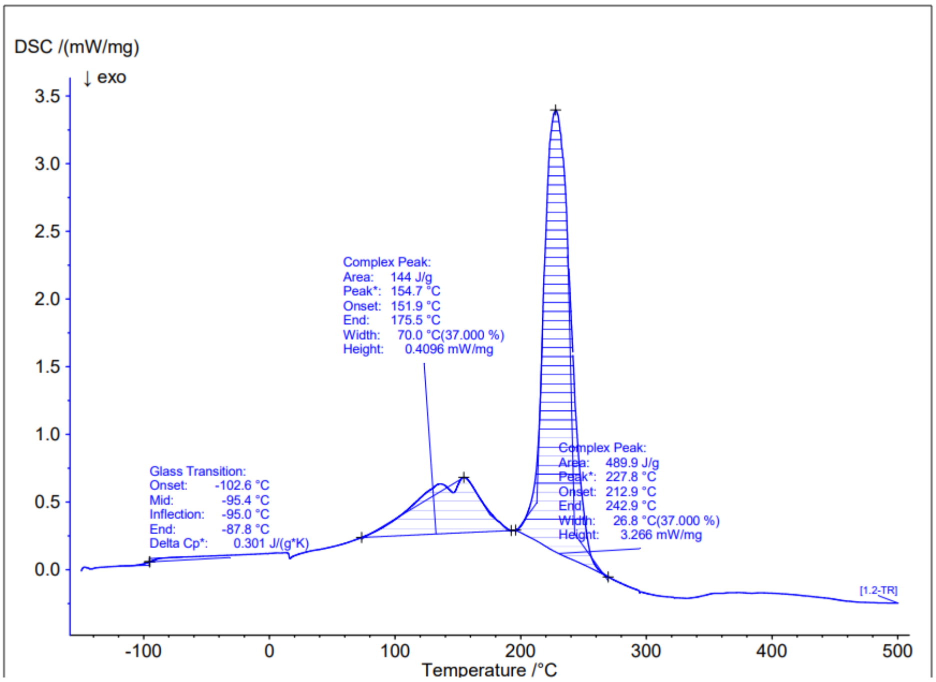

Figure S41. The DSC spectrum of the allyl alcohol oligomer obtained using [VOO(dipic)(2-phepyH)] • H_2O.

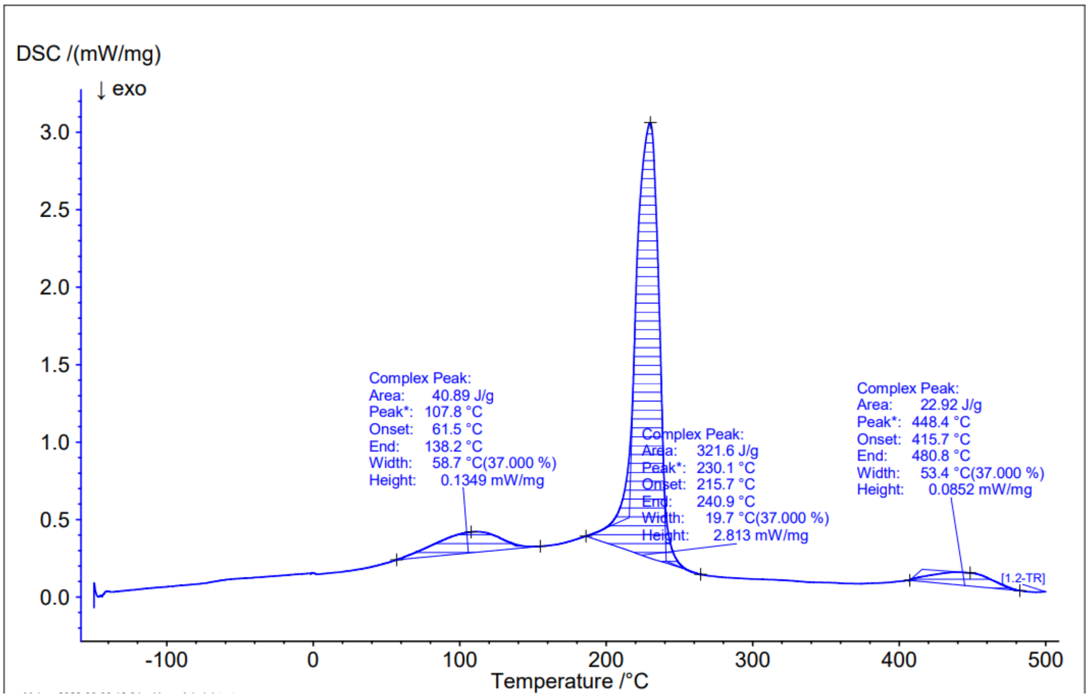

Figure S42. The DSC spectrum of the 2,3-dibromo-2-propen-1-ol oligomer obtained using [VOO(dipic)(2-phepyH)] • H_2O.

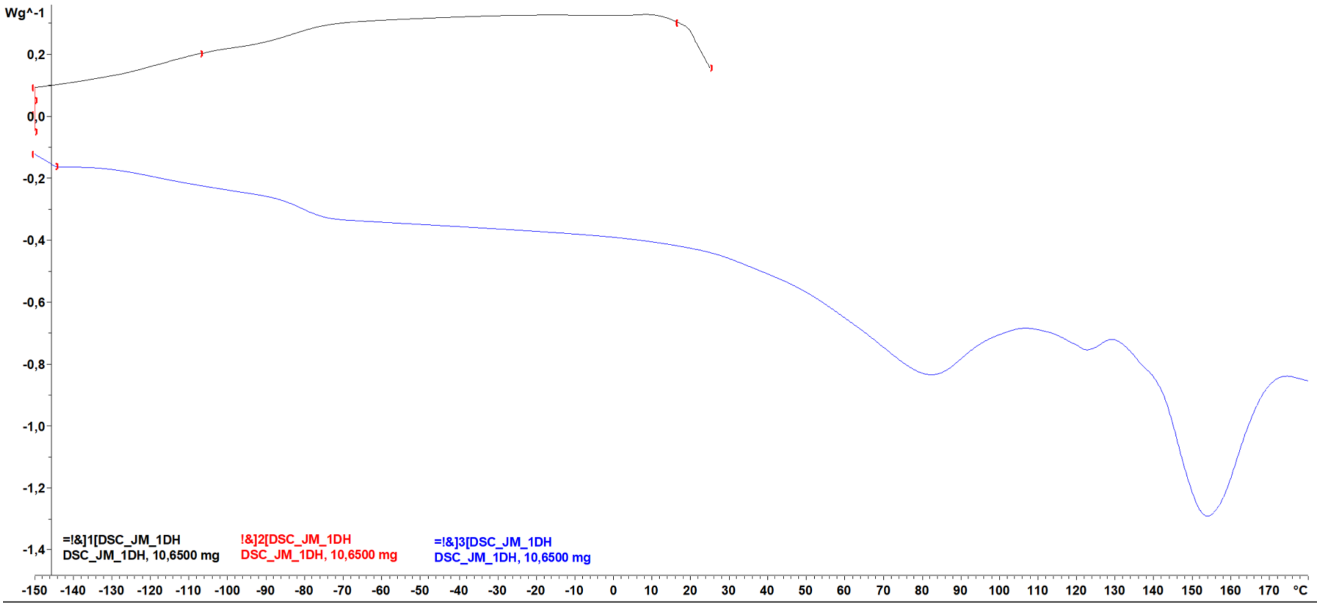

Figure S43. The DSC spectrum of the 3-buten-2ol oligomer obtained using [VO(dipic)(dmbipy)] • 2 H_2O.

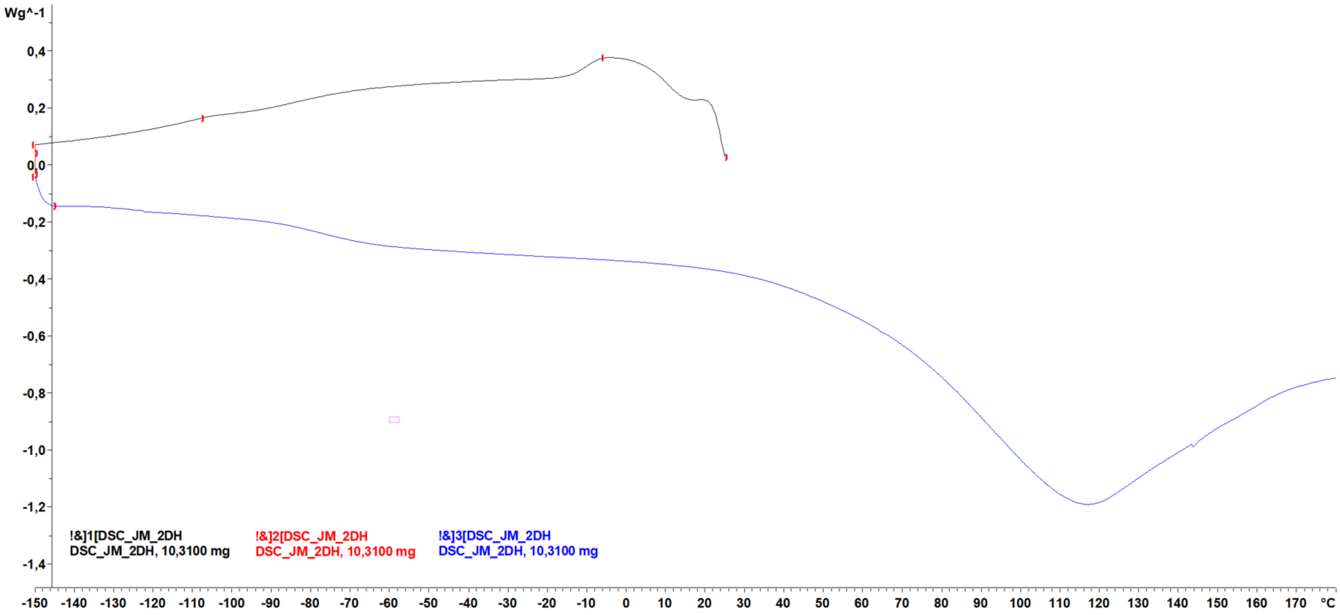

Figure S44. The DSC spectrum of the allyl alcohol oligomer obtained using [VO(dipic)(dmbipy)] • 2 H_2O.

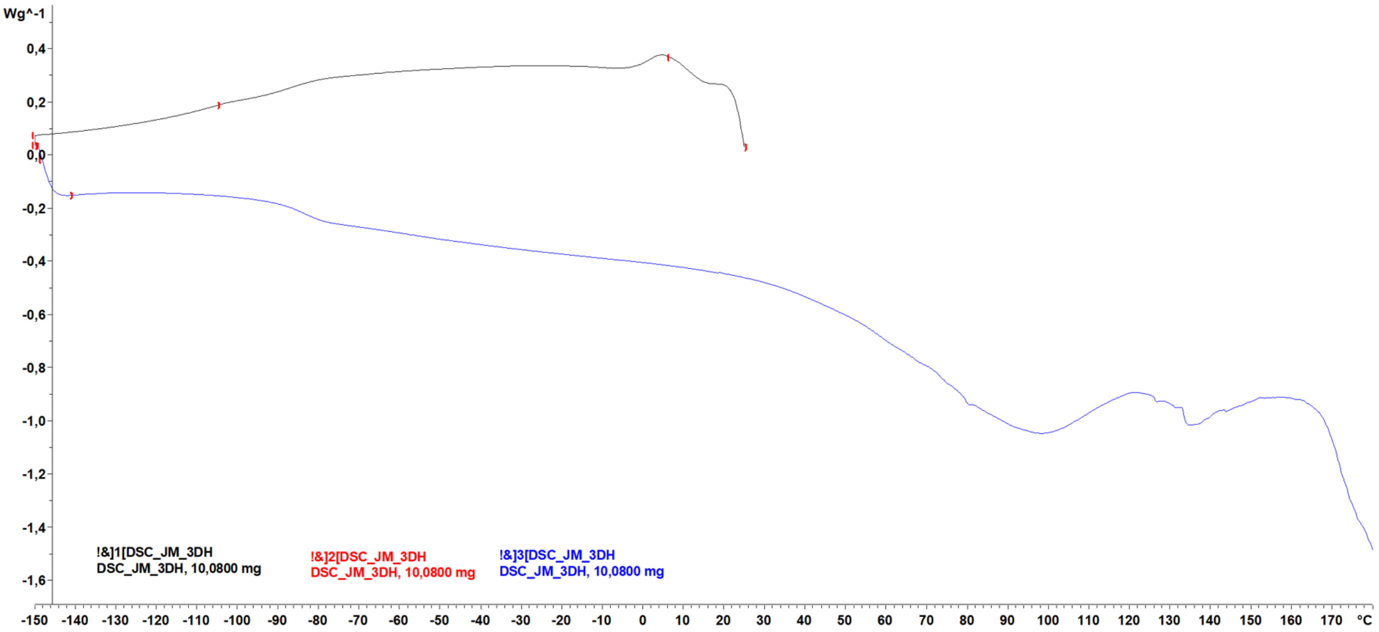

Figure S45. The DSC spectrum of the 2,3-dibromo-2-propen-1-ol oligomer obtained using [VO(dipic)(dmbipy)] • 2 H_2O.

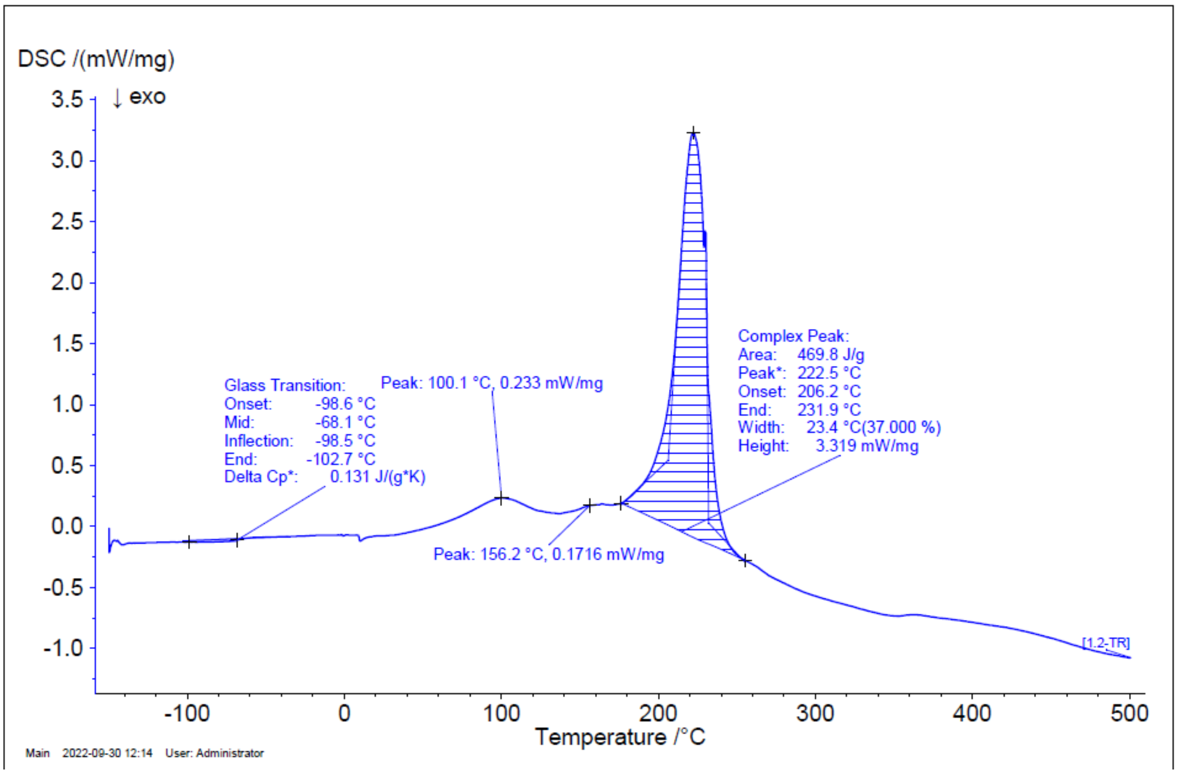

Figure S46. The DSC spectrum of the 3-buten-2-ol oligomer obtained using [VO(ODA)bipy] • 2 H_2O.

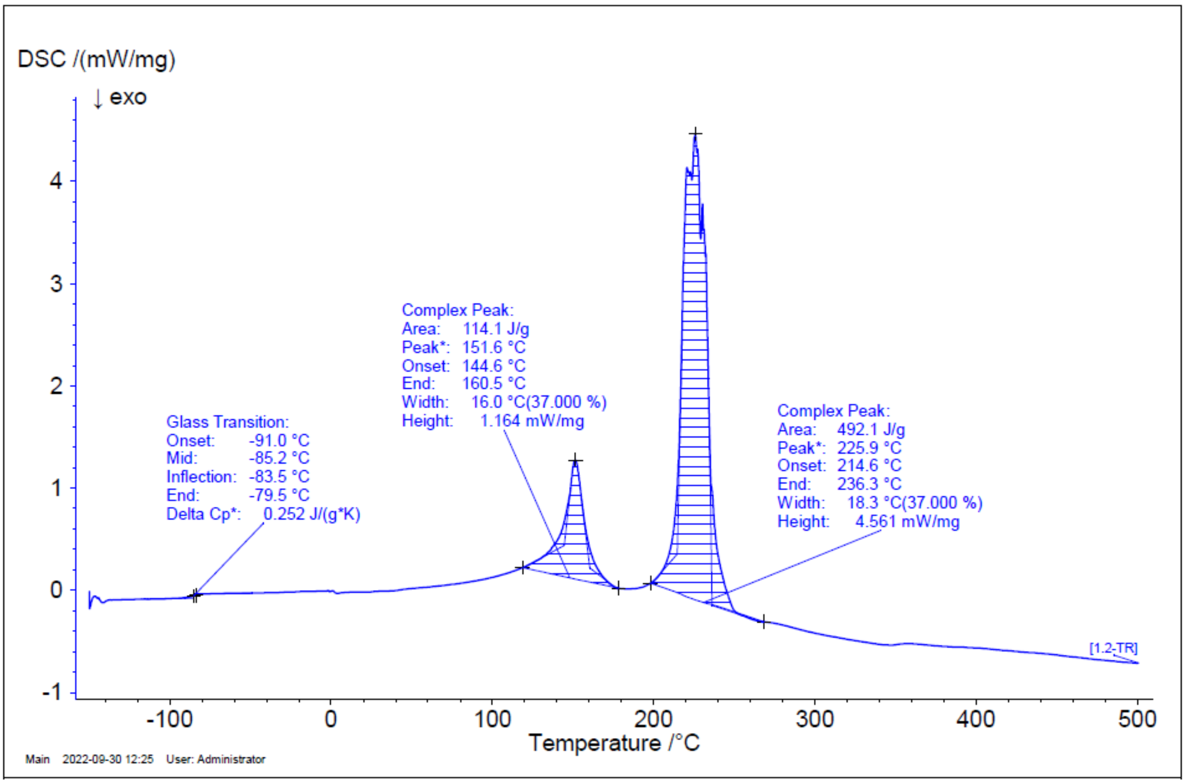

Figure S47. The DSC spectrum of the allyl alcohol oligomer obtained using [VO(ODA)bipy] • 2 H_2O.

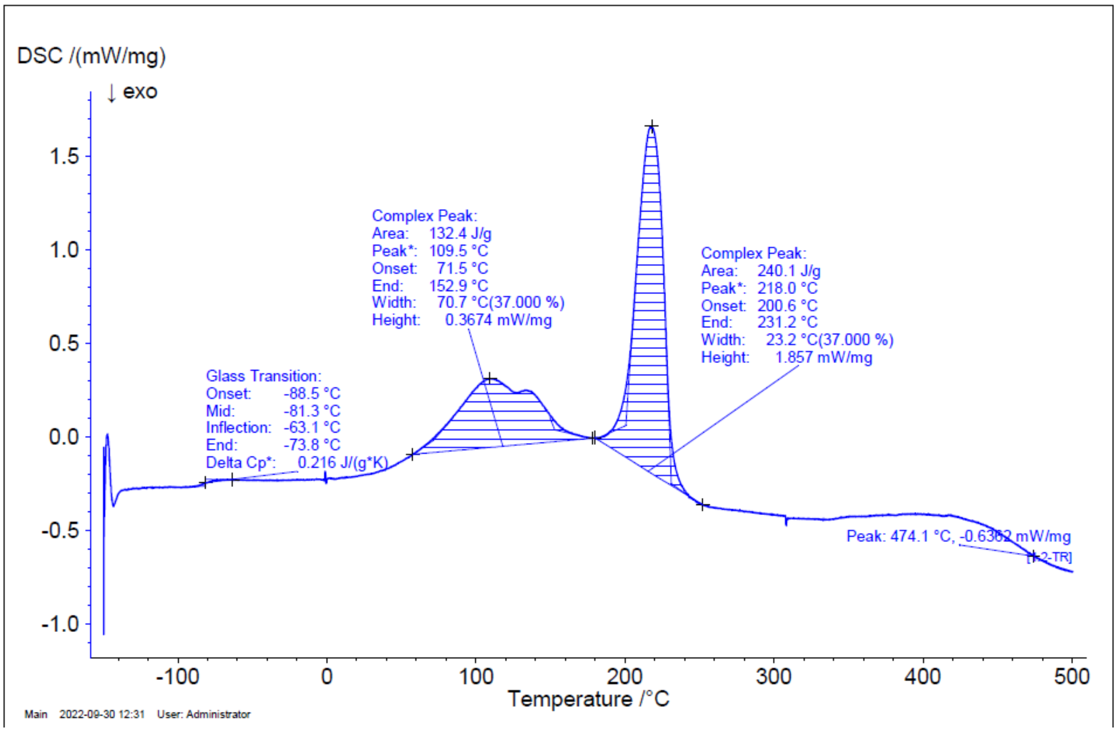

Figure S48. The DSC spectrum of the 2,3-dibromo-2-propen-1-ol oligomer obtained using [VO(ODA)bipy] • 2 H_2O.
